# Pathogenesis and treatment strategies of sepsis-induced myocardial injury: modern and traditional medical perspectives

**DOI:** 10.7150/ijbs.111288

**Published:** 2025-05-15

**Authors:** Wenwen Yang, Yanting Cao, Jiayan Li, Xin Zhang, Xiaoyi Liu, Ye Tian, Liang Shan, Yang Yang

**Affiliations:** 1 Department of Internal Medicine, Shaanxi Provincial People's Hospital, 256 Youyi West Road, Xi'an 710068, Shaanxi, China; 2 Xi'an Key Laboratory of Innovative Drug Research for Heart Failure, Faculty of Life Sciences and Medicine, Northwest University, 229 Taibai North Road, Xi'an 710069, China

**Keywords:** Sepsis, Myocardial injury, Pathogenesis, Modern medicine, Traditional Chinese medicine

## Abstract

Sepsis is a life-threatening organ dysfunction caused by a dysregulated host response to infection. Myocardial injury is a common complication in sepsis patients, which accelerates the progression of sepsis, leading to multiple organ dysfunction and poor prognosis. However, there are still many uncertainties about the characteristics, pathogenesis, treatment, and prognosis of sepsis-induced myocardial injury. While modern medical approaches dominate current clinical management of sepsis-induced myocardial injury, emerging evidence highlights the growing therapeutic potential of traditional Chinese medicine in this field, driven by advances in biomedical research. The integration of these two paradigms holds promise for elucidating the pathophysiological mechanisms and identifying novel therapeutic targets for sepsis-induced myocardial injury, which may accelerate the development of innovative treatment strategies. Therefore, this review comprehensively summarizes the pathogenesis and therapeutic interventions of sepsis-induced myocardial injury from both modern medicine and traditional Chinese medicine perspectives, and critically analyzes the two aiming to provide a valuable reference for researchers' understanding of sepsis-induced myocardial injury.

## 1. Background

Sepsis is a systemic inflammatory response disorder caused by infection, leading to life-threatening multiple organ dysfunction. It is a clinically prevalent critical condition in clinical practice. Approximately 50% of sepsis patients admitted to the Intensive Care Unit (ICU) develop myocardial dysfunction. Furthermore, among sepsis-related fatalities, 70% to 90% of cases involve patients with cardiac dysfunction 1. Myocardial injury is one of the common organ dysfunctions in sepsis, which accelerates the progression of sepsis and is an important factor leading to multiple organ dysfunction syndrome (MODS) and poor prognosis 2. It is characterized by left and/or right ventricular systolic and/or diastolic dysfunction, with elevated levels of myocardial injury markers. Studies have shown that sepsis can cause dysregulation of cardiac response, increase in myocardial suppressors, oxidative reactions imbalance, dysfunction of adrenergic pathways, and damage to myocardial mitochondria. These changes inhibit cardiac energy metabolism and induce myocardial cell death, ultimately leading to cardiac dysfunction. However, the complexity of these pathogenic crosstalk limits the therapeutic value derived from basic and clinical research.

Currently, among the agents for sepsis-induced myocardial injury, vasopressors such as adrenaline and noradrenaline (NA) are employed to increase blood pressure and restore myocardial contractility. However, numerous studies have suggested that vasopressors increase cardiac afterload and cause arrhythmias, potentially worsening cardiac function 3. Additionally, positive inotropic drugs such as dobutamine improve cardiac function parameters in patients with septic shock, but cannot alter organ damage at the late-stage of sepsis 4. Other treatment options include supportive therapies such as antibiotics, or fluid resuscitation. Therefore, there is an urgent need for researchers and medical professionals to continue exploring and developing new targets and drugs with high specificity, minimal side effects, and significant efficacy in treating sepsis-induced myocardial injury. In recent years, traditional Chinese medicine (TCM) has demonstrated unique advantages in the treatment of sepsis and sepsis-induced myocardial injury. For instance, Dahuang Mudan Decoction (DMD) is a traditional Chinese medicinal formula originating from the *Synopsis of the Golden Chamber* with a history spanning thousands of years. Clinically, DMD is commonly employed to treat acute abdominal infections, such as appendicitis 5. According to TCM theory, DMD can be divided into two components: the Huoxue prescription (comprising Moutan Cortex and Natrii sulfas) and the Xiexia prescription (comprising Radix Rhei Et Rhizome, Benincasae semen, and Mangxiao). The Huoxue prescription of DMD has been shown to exert protective effects in septic mice by inhibiting inflammatory and coagulation pathways 6. This prescription counteracts sepsis-associated coagulation dysfunction, thereby exerting beneficial effects during the pathogenesis of sepsis. Therefore, Xuebijing, a compound composed of multiple blood-circulation-activating drugs that have demonstrated significant protective effects in both sepsis and sepsis-induced myocardial injury, has been approved in 2004 by the National Medical Products Administration (NMPA, China) for the treatment of sepsis and MODS 7. However, the complexity of the components in TCM also increases the difficulty of investigating its detailed mechanisms. Consequently, the exploration of active ingredients in TCM has become a significant source for the development of new drugs targeting sepsis-induced myocardial injury.

**Figure 1:**
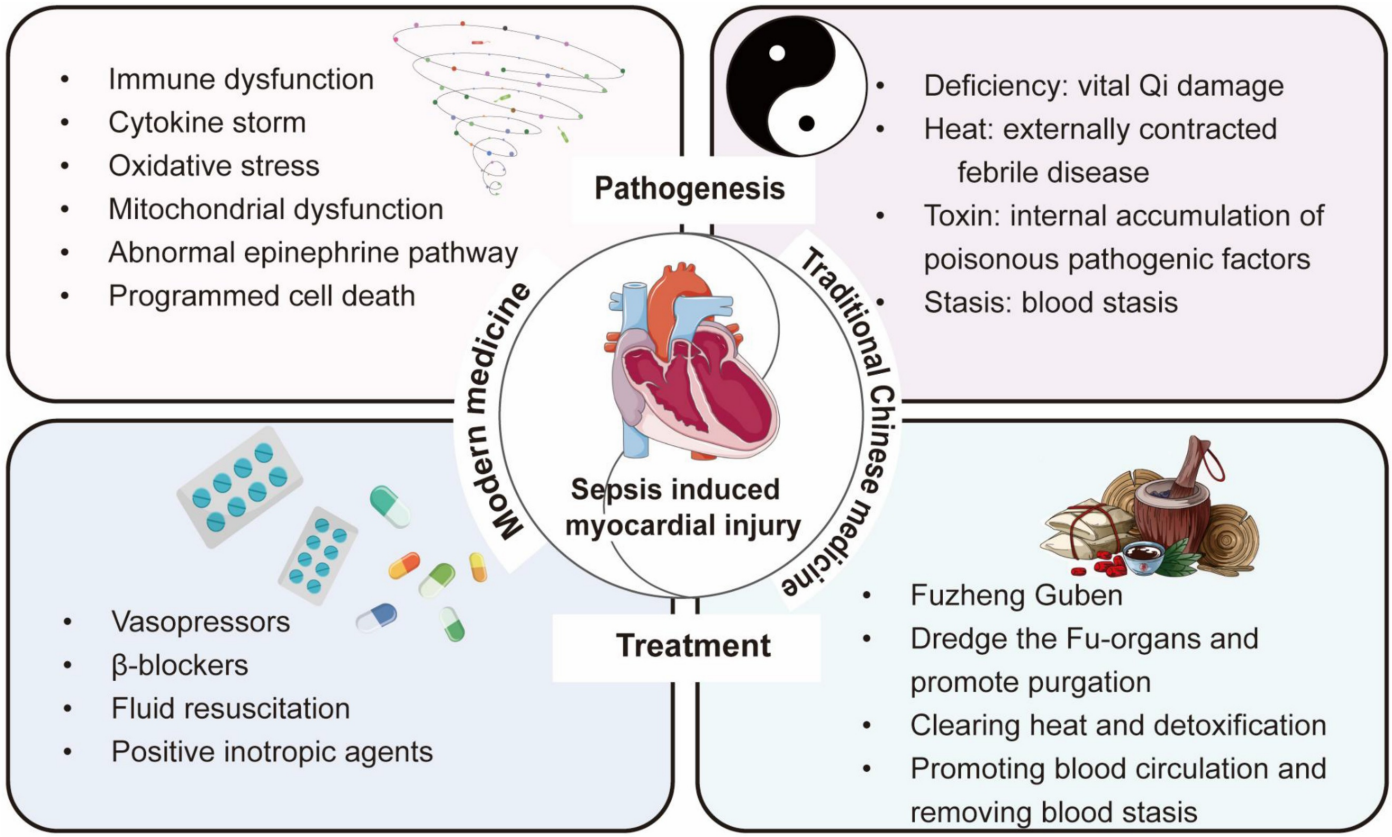
Summary of the pathogenesis and treatment strategies of sepsis-induced myocardial injury from the perspectives of modern medicine and traditional Chinese medicine.

In summary, modern medical treatment strategies place greater emphasis on the “effect”, targeting the direct pathophysiological mechanisms and clinical manifestations of diseases. Modern medicine prioritizes is the use of advanced scientific technologies to precisely identify the etiology and implement targeted therapies. In contrast, TCM focuses more on the “cause”, analyzing the root of conditions such as sepsis and sepsis-induced myocardial injury (deficiency, heat, toxin, and stasis) from a holistic perspective through pattern differentiation and treatment. Despite the significant differences in theory and methodology between the two medical systems, the deepening of interdisciplinary research has gradually led to a complementary relationship between modern medicine and TCM. For instance, in the field of sepsis treatment, scholars have developed clinical practice guidelines for the use of TCM alone or in combination with antibiotics 8. This not only highlights the advantages of integrating modern medicine with TCM but also provides a diversified therapeutic outlook for the treatment of sepsis and sepsis-induced myocardial injury. Therefore, this review comprehensively elaborates on the pathogenesis of sepsis-induced myocardial injury and the advantages and disadvantages of the existing modern medicine treatment methods. Subsequently, we summarize the etiology and pathogenesis of sepsis-induced myocardial injury from the perspective of TCM, as well as the recent progress in TCM therapeutic strategies (**Figure 1**).

## 2. Modern medical insights into sepsis-induced myocardial injury

### 2.1 Overview of sepsis-induced myocardial injury

Sepsis is a life-threatening organ dysfunction caused by a dysregulated host response to infection, with high morbidity and mortality, and has been listed as a global health priority by the World Health Organization 9. Myocardial injury is a common complication of sepsis, with an incidence of over 40%, which accelerates the progression of sepsis, leading to MODS and poor prognosis. In the early stage of sepsis patients with heart damage, the main manifestations are left ventricular systolic and diastolic dysfunction, such as overall longitudinal strain damage to the left ventricle, decreased left ventricular ejection fraction alone or in combination, and abnormal early diastolic peak velocity of the mitral annulus (e') and the ratio of early diastolic peak velocity of mitral flow (E) to e' (E/e') 10, 11; Right ventricular dysfunction, such as impaired free wall strain of the right ventricle; And elevated levels of myocardial injury markers, such as troponin, B-type natriuretic peptide 12, 13. The possible mechanisms of sepsis-induced myocardial injury include immune dysfunction, cytokine storm, abnormal adrenaline pathways, oxidative stress, mitochondrial dysfunction, and programmed cell death. Although researchers have gained a deeper understanding of sepsis-induced myocardial injury, current treatment methods mainly focus on supportive therapies such as early fluid resuscitation and broad-spectrum antibiotics. Interventions targeting the heart, such as the use of vasopressors and inotropes, have been developed. However, controlling the optimal timing and dosage of these drugs remains challenging 14. Therefore, there is an urgent need to develop specific, high-efficacy, low-side-effect treatments for sepsis-induced myocardial injury.

### 2.2 Modern medical pathogenesis of sepsis-induced myocardial injury

#### 2.2.1 Immune dysfunction

**Figure 2:**
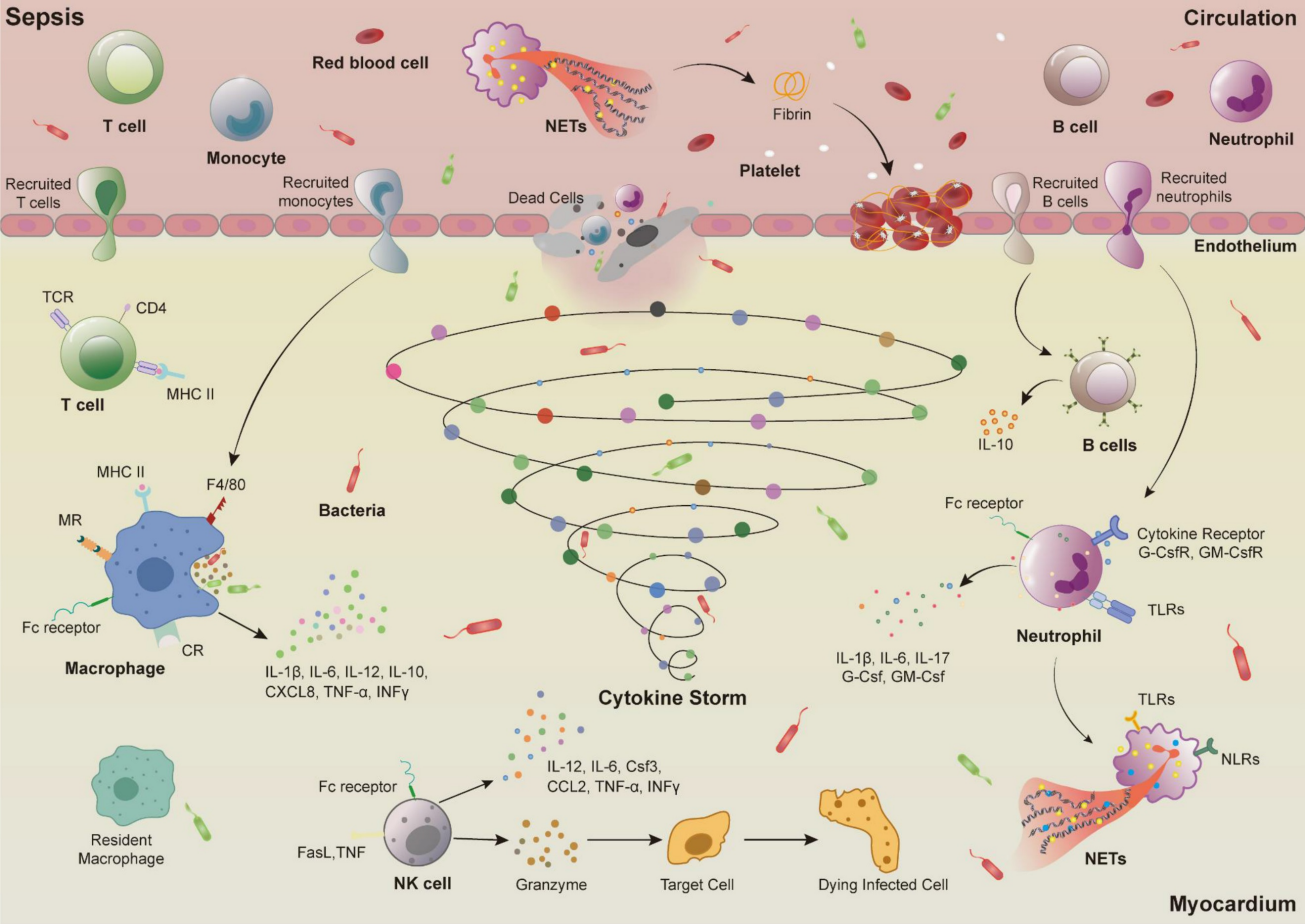
** Immune cell dysfunction in sepsis-induced myocardial injury.** Infectious substances such as bacteria enter the circulation and activate various immune cells. Circulating monocytes are recruited into the myocardial tissue and mature into macrophages, releasing inflammatory cytokines such as IL-1β, IL-6, IL-12, IL-10, CXCL8, TNF-α, INFγ. Neutrophils trapped in blood vessels release NETs, activate platelets, promote fibrin formation, and induce thrombus formation. Activated NETs also stimulate endothelial cells to produce a large number of adhesion molecules, leading to increased permeability of endothelial cells. Neutrophils infiltrate myocardial tissue and release inflammatory cytokines such as IL-1β, IL-6, IL-17, G-Csf, and GM-Csf, and also release NETs and phagocytose pathogens. In addition to secreting inflammatory cytokines such as IL-12, IL-6, Csf3, TNF-α, INF-γ, activated NK cells also secrete cytotoxic proteins such as perforin and granzyme, which directly cause cell and even tissue necrosis.

The inflammatory response caused by immune dysfunction is the main mechanism of sepsis-induced myocardial injury 15. After sepsis occurs, the immune cells in the heart primarily consist of macrophages, monocytes, neutrophils, natural killer (NK) cells, T cells, and B cells 16. Activated immune cells release a large amount of cytokines, which further activate immune cells. This cascade triggers a strong response of the immune system, resulting in excessive inflammatory response and ultimately leading to myocardial damage and cardiac dysfunction (**Figure 2**).

##### (1) Macrophages/monocytes

Around normal myocardial cells, there are some resident macrophages, accounting for 5%—10% of non-myocardial cells in the heart. These macrophages originate from embryos and can clear dead cells or dysfunctional organelles expelled from cells, produce nutrients and immune-related factors, and regulate the electrical conduction of the atrium and atrioventricular node by directly contacting myocardial cells, thereby participating in the regulate of cardiac homeostasis 17, 18. During sepsis, the resident macrophages in the heart can also protect against sepsis-induced myocardial damage by removing dysfunctional mitochondria expelled by cardiomyocytes. Therefore, regulating the number and activity of resident cardiac macrophages may be a potential therapeutic strategy 19. However, activated resident macrophages in the heart and other immune cells can release large amounts of cytokines, recruit monocytes to infiltrate the heart, mature into circulating macrophages, and initiate an excessive inflammatory response 20, 21. Research has found that during sepsis-induced myocardial injury in mice, the number of macrophages in the heart slightly decreases by day 3, but their heterogeneity significantly increases (identifying five subgroups: Mac1-5), making them the most prominent group of cardiac immune cells during sepsis 16. Mac1-like macrophages have similar characteristics to resident macrophages in cardiac tissue 22, while Mac2-5 resemble circulating macrophages in the heart. When the heart is externally disturbed, these types of macrophages express high levels of major histocompatibility complex (MHC) II, presenting invading antigens to T cells, initiating an adaptive immune response, and secreting interferons, chemokines, interleukins (ILs), and other mediators 23-26. These secretions continue to bind to pattern recognition receptors (PRRs) and cellular receptors on activated macrophages 27, forming a broad “signal sensor system” that amplifies and overactivates the immune system, ultimately causing myocarditis, myocardial cell death, and heart dysfunction.

However, the cardioprotective effect of macrophages also exists during sepsis. Studies have shown that when the anti-inflammatory gene interleukin (IL)-10 is specifically knocked out in macrophages, mice exhibit increased levels of cardiac troponin and myocardial cell apoptosis after cecal ligation and puncture injury (CLP). In addition, the number of major histocompatibility complex (MHC) II macrophage subgroups decreases. Additionally, using flow cytometry, researchers found that the number of macrophages in cardiac tissue significantly decreases on the first day of CLP injury, significantly increases on the fourth day, and basically recovers to baseline levels by the ninth week. This suggests that increased macrophages can secrete a large amount of IL-10, enhance the bacteria-clearing ability of macrophages, and thereby provide cardioprotection 28.

##### (2) Neutrophils

Cytokines released by macrophages after activation, in addition to recruiting monocytes, also cause a large number of neutrophils to infiltrate myocardial tissue, inducing neutrophils to release pro-inflammatory signals, produce reactive oxygen species, and generate neutrophil extracellular traps (NETs) to eliminate pathogens 29, 30. Studies have found that co-culturing the serum or plasma of septic patients with neutrophils can induce the production of excessive NETs. During sepsis, NETs can be released along with neutrophils migrating to sites of inflammation, neutralizing and killing invading pathogens. However, excessive release of NETs may further induce inflammation and myocardial damage, exacerbating the development of sepsis-induced myocardial injury 31, 32. Excessive release of NETs during the pathogenesis of sepsis also activates the coagulation pathway. NETs, composed of DNA and histones, activate factor VII to VIIa via their nucleic acids, triggering the coagulation cascade (factor Xa generation, thrombin production, and fibrin formation) 31. Additionally, histones in NETs can activate platelets, further promoting the formation of platelet-neutrophil complexes. These complexes amplify immune and coagulation responses through positive feedback loops, ultimately leading to circulatory dysfunction and organ damage 33. The abundant generation of thrombin and fibrin may lead to microvascular disease, which potentially progresses to disseminated intravascular coagulation, exacerbates multiple organ dysfunction including the heart, and increases the risk of death 34, 35.

In addition to being activated by cytokines and damage-associated molecular patterns (DAMPs), neutrophils also express PRRs including Toll-like receptors (TLRs) and nucleotide-binding oligomerization domain-like receptors (NLRs), which recognize and bind to DAMPs, triggering signalling cascades and NETs release 36. For example, during the development of sepsis, activation of TLR2 on the surface of neutrophils inhibits the activity of C-X-C chemokine receptor 2 (CXCR2) by activating G-protein-coupled receptor kinase 2, leading to impaired chemotaxis of neutrophils. The migration ability is reduced and a large number of neutrophils accumulate within blood vessels. The retained neutrophils stimulate the pro-inflammatory activity of endothelial cells, causing further immune system imbalance. Furthermore, NETs released by accumulated neutrophils stimulate endothelial cells to produce adhesion molecules, induce apoptosis, degrade glycocalyx, increase permeability, leading to capillary leakage, decreased blood pressure, myocardial tissue oedema, and cardiac dysfunction 37, 38.

##### (3) Natural killer cells

NK cells are large granular lymphocytes, with the ability to regulate innate and adaptive immunity, playing an important role in inflammatory responses caused by infections. They can be activated by various PRRs (TLRs, cytokine receptors, etc) on the cell surface 39, 40. During the early stage of sepsis, NK cells are activated by cytokines in the circulation, and subsequently secrete other cytokines such as interferon (IFN)-γ, tumour necrosis factor α (TNF-α), further activating other immune cells (macrophages, neutrophils, and dendritic cells), amplifying the inflammatory response, ultimately leading to multiple organ failure 41. In addition, activated NK cells also secrete cytotoxic proteins such as perforin and granzymes, directly triggering cell and tissue necrosis 42. Therefore, multiple studies have indicated that reducing the number of NK cells during sepsis can reduce organ damage caused by inflammation. For example, in mice with bacterial infection induced by *Escherichia coli* and Group A *Streptococcus*, the use of Asialo-GM1 antibodies to eliminate NK cells decreased the expression of nuclear factor kappa B (NF-κB) and signal transducer and activator of transcription 1 (STAT1), as well as the expression of pro-inflammatory cytokines such as INF-γ, IL-12, and IL-6 in heart, liver, kidney, and lung, ultimately improving the survival rate of septic mice 43, 44. In CLP-induced septic mice, knockout of key genes of NK cells (e.g., β2MKO/αAsGM1) not only reduced systemic inflammatory responses but also enhanced hemodynamics and cardiac contractile function, ultimately reducing mortality 45.

In summary, innate immunity as the first line of defense against infection is rapidly activated upon pathogen invasion. Through PRRs, it recognizes pathogens and activates adaptive immune cells to eliminate pathogens. However, overactivation of immune cells can lead to immune imbalance, ultimately causing tissue and organ damage 46. Therefore, regulating the activity and quantity of immune cells at different timepoints can effectively inhibit excessive inflammatory responses, balance immune dysregulation, and slow down or halt the progression of the disease.

#### 2.2.2 Cytokine storm

Cytokines are some small proteins secreted by immune cells and stromal cells, mediating signal transduction and communication between cells. When sepsis occurs, immune dysfunction leads to the release of large amounts of cytokines from immune cells. These cytokines then trigger the activation of excessive immune cells, forming a vicious cycle. Such cascading amplification may result in uncontrolled hyperinflammation, cellular damage, and organ failure, collectively termed a “cytokine storm”. 47 Cytokines that play a role in sepsis-induced myocardial injury mainly include interleukins (ILs), chemokines, colony stimulating factors (CSFs) and TNF (**Table 1**).

##### (1) Interleukins

Interleukins (ILs) are the most important cytokines released in the process of sepsis, mainly secreted by leukocytes (immune cells) and endothelial cells, mediating the activation, proliferation, movement, and/or death of immune cells. They can be classified into pro-inflammatory and anti-inflammatory categories based on their functions. ILs such as IL-1β, IL-6, IL-12, IL-17 and IL-18 play a crucial role in sepsis-induced myocardial injury.

IL-1β and IL-18 are products after the activation of inflammasomes like nucleotide-binding oligomerization domain-like receptor protein 3 (NLRP3). In CLP-induced septic mice, activated inflammasomes release substantial IL-1β, which binds to IL-1R on cardiomyocytes and reduces myosin heavy chain protein content by regulating autophagy and lysosomal pathway-related molecules. This leads to decreased contraction and relaxation functions of myocardial cells, ultimately resulting in cardiac dysfunction accompanied by myocardial atrophy 48. In addition, studies have found that knocking out the IL-1β gene alone cannot inhibit lipopolysaccharide (LPS)-induced myocardial injury. However, simultaneous inhibition of IL-18 expression significantly improves the reduction of LPS-induced cardiac fractional shortening (FS) rate in mice, inhibits the elevation of the heart function marker creatine kinase isoenzyme MB (CK-MB), and improves cardiac function 49.

IL-6 is produced by almost all immune cells, playing a central role in the host defence system. During the inflammatory response, IL-6 signalling is not transmitted through the classical membrane receptor binding. Instead, it binds to the soluble IL-6 receptor (sIL-6R) (sIL-6R-IL-6) as a complex and then interacts with the membrane-bound signalling molecule gp130 on the cells to initiate intracellular signal transduction. Therefore, many cells that do not express IL-6R can also be stimulated by the sIL-6R-IL-6 complex to enhance signal transduction 50. A recent clinical study has found that inhibiting sIL-6R activity improves the survival rate and prognosis of sepsis patients, offering for sepsis treatment 51. In addition, in basic research, inhibiting transcriptional activator STAT3 downregulates the expression of IL-6 and gp130, thereby reducing the level of cardiac inflammation, endoplasmic reticulum stress, and mitochondrial dysfunction in CLP- or LPS-induced septic mice, and improving cardiac function 52, 53. Interestingly, research has also shown that IL-6 is involved in the antioxidant stress pathway 54. In the early stage of LPS-induced sepsis, IL-6 supplementation promotes the expression and nuclear translocation of nuclear factor E2-related factor 2 (Nrf2), thereby maintaining the redox homeostasis of myocardial cells. It plays a beneficial role in the early myocardial injury during LPS-induced sepsis 55.

IL-12 is a main molecule in the differentiation of T cells and the induction of IFN-γ, playing an important immunomodulatory role in sepsis-induced myocardial injury. In CLP-induced septic mice, the expression of IL-12 p35 in myocardial tissue is increased, leading to decreased ejection fraction (EF) and FS levels and impaired cardiac function 56. However, some researchers have found that elevated IL-12 p40 exerts anti-inflammatory effects by regulating NF-κB signalling pathways in the LPS-induced septic mice, thereby improving cardiac function 57. IL-17 (also known as IL-17A) is produced by IL-23-stimulated Th17 T cells after sepsis and induces the synthesis of various cytokines such as IL-1β, IL-6, and TNF-α. The secretion of IL-17 is chronically increased in septic mice, which may be related to the impaired function of CD4^+^ T cells (Th1, Th2 and Th17 subsets) 58. However, studies have also indicated that IL-17 plays a protective role during the progression of sepsis. Specifically, IL-17 gene knockout in CLP mice exacerbates inflammatory mediators levels and increases mortality rates 59.

The roles of IL-12 and IL-17 in sepsis-induced myocardial injury exhibit duality. In addition to the different structural and functional characteristics of the cytokines, it may also be because the pathogenic mechanisms of CLP and LPS-induced sepsis models are not completely identical. Moreover, variations in experimental time points may lead to differential IL production under specific inducing factors, resulting in divergent effects at different stages. Therefore, particularly in fundamental experimental studies, it is imperative to objectively analyze and systematically summarize the regulatory patterns of inflammatory responses based on their divergent expression profiles.

##### (2) Chemokines

Chemokines are a class of soluble small molecules that activate and recruit immune cells by binding to specific G protein-coupled receptors. Unlike other cytokines, chemokines exhibit strong cell specificity. C-C chemokine ligand 2 (CCL2), C-X-C chemokine ligand 8 (CXCL8)/IL-8, and CXCL2 have been widely studied in sepsis-induced myocardial injury. CCL2, also known as monocyte chemo-attractant protein-1 (MCP-1), is an important molecule for monocyte chemotaxis, mediating various pro-inflammatory factors 60. Elevated CCL2 levels in the serum of sepsis patients and animal models correlate closely with sepsis-related mortality 61. The expression of CCL2 is also elevated in the myocardial tissue of CLP- and LPS-induced sepsis animals 62. CXCL8, one of the most intensively studied chemokines, is significantly elevated in the serum of septic patients with concomitant heart failure than in patients with sepsis alone. High serum levels of CXCL8 are associated with reduced cardiac EF, cardiac output (CO), and stroke volume (SV), suggesting a relationship between CXCL8 and the sepsis-induced heart failure 63, 64. CXCL2, also known as macrophage inflammatory protein-2 (MIP-2), is associated with the severity of sepsis. Under pathological conditions, CXCL2 is induced by cytokines such as IL-17, NF-κB, and IL-1β 65. In CLP- and LPS-induced septic mice, inhibition of CXCL2 reduces neutrophil recruitment and myocardial cell pyroptosis, alleviating sepsis-induced cardiac dysfunction 66, 67.

##### (3) Colony stimulating factors

Among the various growth factors secreted during sepsis, the CSFs play a particularly significant role in driving the cytokine storm storm. CSFs synergizes with other cytokines to promote bone marrow cell differentiation/proliferation, activate immune cells (e.g., granulocytes, macrophages), and enhance cytokine synthesis, collectively driving cytokine storm progression 68. Studies have shown that knocking out granulocyte CSF (G-CSF, also known as Csf3) can reduce the level of serum myoglobin induced by LPS in mice, improve heart function, and increase the survival rate. This protection may stem from attenuated neutrophil infiltration in Csf3 knockout mice, which lowers inflammatory cytokine secretion post-LPS stimulation and ultimately suppresses cardiac hyperinflammation 69.

##### (4) Tumor necrosis factors

In the 1980s, researchers discovered that macrophages can secrete a soluble small molecule, named TNF-α, which significantly inhibits tumour growth in mice. As research progressed, it was found that TNF-α inhibits cardiomyocyte contraction by disrupting L-type calcium channel-mediated calcium influxand impairing calcium transients, and TNF-α antibody therapy preserves myocardial function in septic animals and patients. These results indicate that TNF-α is an important myocardial inhibitory factor. Additionally, the myocardial suppression mechanism caused by TNF-α also includes inducing myocardial cell toxicity, promoting myocardial cell apoptosis, and activating oxidative stress pathways 70. The characteristic manifestation of sepsis patients is a significant increase in TNF-α levels in circulation 71. Several basic studies also suggest that inhibiting TNF-α expression can alleviate myocardial damage caused by sepsis in animal models 72, 73.

Above all, cytokine storms involve downstream targets with pleiotropic effects, and their biological activities trigger complex, overlapping signaling pathways. While appropriate cytokine production protects against pathogens by resolving inflammation, excessive mediators during hyperinflammation cause multiorgan damage 74.

#### 2.2.3 Oxidative stress

Under the inflammatory conditions of sepsis, enhanced oxidative activity in cardiomyocytes clears pathogens or repairs endothelial damage but concomitantly generates reactive oxygen species (ROS), such as superoxide anions (·O_2_^-^), hydrogen peroxide (H_2_O_2_), and hydroxyl radicals (·OH). Cardiomyocytes contain abundant mitochondria, accounting for approximately 30% of the cellular volume. The mitochondrial electron transport chain (ETC), which transfers H⁺ and electrons, serves as the primary site for ROS generation in cardiomyocytes. ETC complexes I, II, and III contain specific sites where electron leakage prematurely reduces oxygen, forming ROS 75. During sepsis, the elevated NADH/NAD⁺ ratio increases the fully reduced flavin mononucleotide (FMN) at Complex I, providing electrons to oxygen to generate ·O_2_^-^. Additionally, the ubiquinone-binding site within Complex I, which mediates ubiquinone reduction, also regulates ·O_2_^-^ generation. This occurs through incomplete ubiquinone reduction, forming unstable semiquinone radicals that serve as sources of ·O_2_^-^ 76. Complex II contains at least two ubiquinone-binding sites and a flavin adenine dinucleotide (FAD) cofactor that facilitates ·O_2_^-^ production. The FAD-derived ·O_2_^-^ may originate from either FADH₂ auto-oxidation or FADH⁻ semiquinone auto-oxidation 77. In Complex III, ·O_2_^-^ generation is mediated by unstable semiquinone intermediates. Furthermore, the low-potential heme b accepts an electron from unstable semiquinone, contributing to ·O_2_^-^ production in Complex III 78. ROS generated by the ETC are rapidly converted to hydrogen peroxide (H₂O₂) via manganese superoxide dismutase. In the presence of glutathione (GSH), H₂O₂ is further reduced to water by glutathione peroxidase (GPX).

Under physiological conditions, moderate ROS levels participate in cellular processes including growth, differentiation, DNA synthesis, stress responses, and metabolism. Excessive ROS are effectively neutralized by endogenous antioxidant systems. However, during sepsis, the antioxidant capacity is insufficient, with decreased activities of superoxide dismutase (SOD) and catalase (CAT), as well as reduced GSH, leading to oxidative stress. The circulating ROS then stimulates ROS generation in endothelial cells, forming a malignant cycle of free radicals, causing DNA damage, mitochondrial ultrastructural and functional alterations, cardiomyocyte injury, and ultimately organ dysfunction such as cardiac failure 79. Nie et al. found that the long non-coding RNA MCM3AP-AS1 expressed in cardiac tissue can enhance SOD and glutathione peroxidase (GSH-Px) levels by inhibiting the expression of miR-101-3p in mice with CLP-induced sepsis myocardial injury, thereby enhancing antioxidant capacity, cardiac function 80. Additionally, activating the expression of deacetylase 3 in LPS-induced sepsis cell and animal models promotes myocardial mitochondrial biosynthesis, increases SOD activity, and suppresses ROS production. This further inhibits oxidative stress and mitochondrial autophagy, improves levels of cardiac function markers cardiac troponin I (cTnI), CK-MB, and lactate dehydrogenase (LDH), and enhances EF and FS cardiac function parameters, thus alleviating sepsis-induced myocardial injury 81.

In addition, reactive nitrogen species, such as peroxynitrite (ONOO^-^), are generated by the reaction of nitric oxide (NO) with oxygen free radicals in infected cardiac tissue. ONOO^-^, a toxic mediator of NO, directly damages nucleic acids, lipids, and proteins, disrupts the mitochondrial respiratory chain, induces cell death, and impairs cardiac function 82, 83. During sepsis, NO is produced by activated immune cells such as macrophages and neutrophils, and many cytokines can also produce large amounts of NO by activating the transcription factor NF-κB and inducible nitric oxide synthase (iNOS). Excessive NO plays a central role in cardiac contractile dysfunction in septic shock 79. Therefore, extensive basic research has found that inhibiting iNOS activity or NO production effectively mitigates oxidative stress and improve cardiac function in sepsis animals 84, 85.

#### 2.2.4 Mitochondrial dysfunction

Mitochondria are the central hub of signal transduction and cellular metabolism, as well as the main source of adenosine triphosphate (ATP). Under physiological conditions, the heart requires well-structured and functional mitochondria to maintain energy supply for cardiac contraction. However, in the inflammatory environment of sepsis, mitochondrial oxidative phosphorylation (OXPHOS) are disrupted, biogenesis is inhibited, ATP production becomes insufficient, and mitochondrial autophagy is exacerbated. These perturbations collectively lead to cardiomyocyte death, ultimately resulting in cardiac dysfunction and life-threatening systemic consequences.

##### (1) Mitochondrial morphology and structure damage

In patients with sepsis, the integrity of mitochondrial structure plays a crucial role in their prognosis and the development of myocardial dysfunction. One study has examined the myocardial tissue of septic patients who died of myocardial dysfunction (using cardiac transplant recipients and brain-dead donors as controls) via electron microscopy. The results showed that septic hearts exhibited a higher proportion of swollen mitochondria. Furthermore, mitochondria displayed vacuoles of varying sizes, widened cristae gaps, collapsed inner membranes, and significant translocation of connexin 43 (CX43) to the cardiomyocyte membrane periphery—a hallmark of myocardial injury. These morphological alterations correlate with cellular damage and cardiac dysfunction 86. In rats' hearts treated with LPS, mitochondrial swelling, cristae disruption, and vacuolization of the mitochondrial matrix were observed. At the same time, indicators of mitochondrial function such as oxygen consumption, ATP synthesis rate, and mitochondrial respiratory chain complex activity were inhibited 87. In addition, decreased mitochondrial membrane potential and increased mitochondrial fragmentation are found in LPS-induced septic mice and cardiomyocytes, and this process is associated with the interaction between dynamin-related protein 1 (DRP1) and mitochondrial fission protein 1. Inhibiting this process can improve mitochondrial activity and cardiac function in sepsis 88.

Furthermore, mitochondria are highly dynamic organelles, maintaining its normal physiological morphology, quantity, size, and function through division, fusion, and motility. Mitochondria separate damaged components from the healthy physiological network through asymmetrical division and degrade them through mitophagy, which contributes to mitochondrial quality control. However, under pathological conditions of sepsis, excessive mitochondrial division and impaired fusion promote mitochondrial fragment formation, and decrease mitochondrial membrane potential, and result in leakage of pro-apoptotic proteins into the cytoplasm, culminating in cardiomyocyte death 89. Multiple studies have shown that in LPS-induced septic mice exhibit uncontrolled mitochondrial fission in cardiomyocytes, generating excessive fragments and activating caspase-dependent apoptosis. Inhibiting the activity of the key molecule DRP1 that regulates mitochondrial division can effectively suppress mitochondrial division, increase cardiomyocyte viability, improve cardiac dysfunction, and reduce mortality 88, 90, 91. Similarly, in CLP-induced septic rats by, Juana et al. found that thioredoxin 1 overexpression significantly inhibits the expression of DRP1, upregulates mitochondrial transcription factor A (TFAM) and uncoupling protein 2 (UCP2), and enhances cardiac contractility 92.

##### (2) Mitochondrial permeability transition pore opening

The mitochondrial permeability transition pore (mPTP) protein complex is a non-specific channel between the inner and outer mitochondrial membranes, which mediates the permeability transition of the mitochondrial inner membrane. According to the chemiosmotic hypothesis, proton extrusion by the electron transport chain generates an electrochemical gradient across the inner membrane, driving ATP synthesis. Thus, the inner membrane remains impermeable under physiological conditions. However, during sepsis, Ca^2+^ overload, oxidative stress, ATP depletion and other factors lead to prolonged mPTP opening, which increases the permeability of the mitochondrial inner membrane and causes substances from the intermembrane space to transfer to the mitochondrial matrix. Subsequently, mitochondrial inner membrane potential imbalances, OXPHOS disorders, ATP synthesis decreases, mitochondria swell, cristae structures dissolve, and ultimately rupture of the outer membrane leads to the release of cytochrome C and other pro-apoptotic proteins, initiating cell death 93, 94. Hu et al. found that activation of mitochondrial aldehyde dehydrogenase can inhibit the generation of ROS, block mPTP opening, and alleviate LPS-induced myocardial cell damage in mice 95. Furthermore, Cao et al. proposed that supplementing nicotinamide mononucleotide inhibits mPTP opening by increasing NAD^+^ level and clearing excess ROS, and ultimately suppress LPS-induced inflammation and apoptosis in cardiomyocytes and mouse hearts 96. In addition, the specific mPTP inhibitor cyclosporine A enhances mitochondrial function, improve cardiac function, and reduce mortality in CLP and LPS models of sepsis 97.

##### (3) Energy metabolism abnormalities

About 70% of the energy required by the heart is produced by fatty acid oxidation, with the rest mainly derived from glucose oxidation. The process of energy production involves mitochondria using the reduced electron carriers (FADH_2_ and NADH) generated by fatty acid β-oxidation and glucose metabolism as substrates, transferring the electrons to ATP synthase on the inner mitochondrial membrane for ATP production. During sepsis, activation of the sympathetic nervous system releases a large amount of catecholamines to stimulate β-2 adrenergic receptors, promoting glycolysis to produce large amounts of pyruvic acid and H^+^. However, the structure and quantity of mitochondria are damaged, and the OXPHOS capacity is decreased, causing pyruvic acid to generate large amounts of lactate in the cytoplasm instead of entering the mitochondria to produce energy. Myocardial cells lack energy supply and cannot function properly, ultimately leading to heart dysfunction 98. Additionally, during sepsis, the levels of triglycerides and free fatty acids in the blood increase, while the intake of fatty acids and other lipids by tissues such as the heart decreases. Mitochondrial dysfunction also inhibits fatty acid oxidation and OXPHOS, which increases the consumption of ATP and leads to energy deficiency in the heart 84, 99. Studies have found that the expression of fatty acid binding proteins in cardiomyocytes is decreased in LPS-induced septic myocardial injury animals, as well as the expression of transcription factors closely related to fatty acid oxidation such as peroxisome proliferator-activated receptor (PPAR), and PPARγ coactivator 1 (PGC-1) are reduced. Suppressing these expressions inhibits the mobilization and oxidation of fatty acids in the myocardium while increasing the expression of these molecules can improve cardiac function and increase the survival rate of septic animals 100, 101.

In addition, the damage of ETC is also one of the important reasons for the lack of heart energy. Mitochondria are the main sources of ROS and reactive nitrogen species, and they are involved in cell signalling under normal physiological conditions. However, when sepsis occurs, levels of ROS and others increase and Fenton reaction occurs between H_2_O_2_ and the iron-sulfur complexes in the ETC, leading to impaired ETC function 102. Beyond direct impairment of ETC proteins, mitochondrially-derived ROS also oxidize lipids, proteins, and DNA. Notably, mitochondrial DNA (mtDNA) is particularly vulnerable to ROS-induced damage due to the absence of protective histones. Furthermore, the content of oxidatively modified bases in mtDNA is typically 10-20 times higher than in nuclear DNA 102. Mitochondrial NO competes for binding sites with oxygen in Complex IV, reducing OXPHOS efficiency. Additionally, the reaction of NO with oxygen free radicals generates a large amount of ONOO^-^, which causes nitration of tyrosine residues in ETC complex proteins, irreversibly reducing ATP synthesis 103, 104. After CLP injury, the number of mitochondria in mouse myocardial tissue is decreased, and the activity of Complex III is also inhibited, leading to impaired mitochondrial ETC and OXPHOS processes 105. A septic shock model in young pigs also showed that after 24 hours of sepsis onset, the functions of mitochondrial Complexes II and IV are impaired, blood lactate levels are increased, the contractility of young pig hearts is decreased, and heart rate is accelerated 106. In addition, in septic mice induced by CLP, the activity of Complex IV in the electron transport chain is reduced, and the expression levels of its subunits are also decreased. Timely administration of exogenous cytochrome c oxidase (the main component of Complex IV) significantly restores mitochondrial activity and improves heart function 107. In conclusion, the heart highly depends on a large amount of ATP to maintain its normal contraction and relaxation functions, and once energy is lacking, heart function may also be compromised.

#### 2.2.5 Abnormal adrenergic pathway

The inflammatory response during sepsis rapidly activates the sympathetic nervous system (SNS). Activation of the hypothalamic-pituitary-adrenal axis enhances catecholamine release (e.g., NA and adrenaline) from postganglionic sympathetic neurons and the adrenal medulla 108. SNS branches are present in immune organs such as the thymus, spleen, bone marrow, and lymph nodes, reflecting its profound interplay 109. During sepsis, the excessive release of catecholamines makes it difficult to maintain balance in immune regulation and cardiovascular function support. Although NA is commonly used as a vasopressor in the treatment of septic shock, there are still many doubts. For example, 1) NA may cause immune imbalance, further worsening infection and sepsis progression; 2) Higher concentrations of endogenous or exogenous catecholamines in ICU patients are associated with poorer clinical outcomes; 3) Adrenaline stimulation inhibits the activity of innate and adaptive immune cells 110. Therefore, inhibiting the expression of adrenaline and adrenergic receptor signalling under appropriate conditions is a new perspective for treating patients with sepsis.

In the cardiovascular system, there are abundant adrenergic receptors (including α-ARs and β-ARs), which are prone to pathological overstimulation by the SNS. It leads to apoptosis of presynaptic neurons and glial cells in the cardiac autonomic centre, the impairment of contractile function, tachyarrhythmia, and the decrease of the sensitivity of the heart to endogenous catecholamines. In CLP-induced septic rats and LPS-treated cardiomyocytes, the expression of β3-AR is upregulated. Furthermore, β3-AR antagonists (SR59230A) significantly reduce the maximum rate of left ventricular pressure rise in CLP induced septic rats, thereby preserving myocardial contractile function during sepsis 111. Additionally, the expression of β1-AR is increased in LPS-treated mouse cardiomyocytes. β1-AR agonists increase intracellular Ca^2+^ levels, decrease anti-apoptotic protein Bcl2, and promote pro-apoptotic protein Bax mitochondrial translocation, ultimately inducing apoptosis 112. A randomized clinical trial found that the β-blocker esmolol can lower heart rate, increase left ventricular work index, and reduce 28-day mortality in patients with septic shock 113.

The activity of α-ARs in the heart is also crucial for maintaining normal cardiac function and is activated during acute sympathetic stress. In the LPS-induced sepsis model, blocking α2-AR can suppress the expression of myocardial TNF-α and iNOS, reduce apoptosis, and improve cardiac dysfunction 114. In addition, in porcine appendicitis-induced sepsis, the α1-AR agonist methoxamine can amplify the inflammatory response by activating the Gq protein-coupled receptor and the second cell messenger phospholipase C (PLC), thereby exacerbating organ damage 115, 116. Cardiac inflammation is an important pathological process of excessive catecholamine-induced myocardial damage 117, 118. In C57 mice, activation of α1-AR with phenylephrine can activate the Kir2.1/NF-κB/NLRP3 axis, promoting macrophage infiltration and pro-inflammatory cytokine release (IL-1β, IL-6, IL-18), thereby exacerbating cardiac inflammation 119. Therefore, regulating adrenergic receptor signaling in cardiac muscle cells can provide new therapeutic targets for sepsis-induced myocardial injury.

#### 2.2.6 Programmed cell death

##### (1) Apoptosis

Apoptosis is the first discovered programmed cell death pathway, characterized by nuclear fragmentation, chromatin condensation, and formation of apoptotic bodies. There are mainly three mechanisms of apoptosis in cells. The first is the mitochondrial pathway, that is, under cellular stress, the anti-apoptotic protein Bcl2 decreases, leading to an increase in pro-apoptotic factors Bak and Bax proteins. Assembly of these effectors into a large complex increased mitochondrial membrane permeability and release of cytochrome c, which then combines with apoptotic peptidase activating factor 1 (APAF-1) to form apoptotic bodies, recruiting and activating Caspase9. The second is the death receptor pathway, which activates Caspase8 by triggering death receptors (such as TNF-R1, FAS/CD95) on the cell membrane. Caspases generated by both pathways eventually induce the activation of effector proteins Caspase3 and Caspase7, which promotes the exposure of phosphatidylserine on the outer membrane of damaged cells (a signal for phagocytes to “eat me”) and induces apoptosis 120. Several studies have shown that reducing the expression of Bax, Caspase3, and Caspase9, and promoting the expression of Bcl2 can inhibit cardiomyocyte apoptosis, and improve the heart function induced by CLP- or LPS-induced sepsis in animals 121-123. However, there is currently limited research on the death receptor cell apoptosis pathway in sepsis-induced myocardial injury. In some studies, it was found that Caspase8 and Bax are activated together to induce cardiomyocyte apoptosis 124, 125. The third mechanism of apoptosis is the endoplasmic reticulum (ER) stress pathway. When sepsis leads to intracellular environmental imbalance, there is excessive and sustained ER stress due to misfolding or increased number of unfolded proteins, resulting in a large amount of ER-related molecules and cell apoptosis 126. The expression of ER-related chaperone protein glucose regulator protein 78 (GRP78) is significantly increased in a rat model of septic myocardial injury constructed by CLP. The expression of key molecules CHOP and Caspase12 in the ER stress-mediated apoptosis pathway are also significantly increased, suggesting that ER stress-mediated cell apoptosis is also involved in the process of sepsis-induced myocardial injury. After the inhibition of GRP78, CHOP, and Caspase12, the number of cardiomyocyte apoptosis is decreased, the level of EF and FS are increased, and the cardiac function is restored 127, 128.

##### (2) Necroptosis

In 2005, Degterev and his team showed that serine/threonine-protein kinase 1 (RIPK1) can be inhibited to prevent TNF-induced fibroblast death, demonstrating the controllability of RIPK1-mediated cell death 129. Therefore, a novel non-apoptotic programmed cell death pathway, necroptosis, has been identified. Its activation process is mainly mediated by TNF-R1 or TLR and regulates the downstream RIPK1 and RIPK3 to form heterologous complexes. RIPK3 activates mixed-lineage kinase domain-like (MLKL) and translocates to the cell membrane. Then Ca^2+^ influx is initiated, leading to cell swelling and membrane rupture, secretion of a large number of DAMPs, and aggravation of the inflammatory response 130. Studies have found that in the CLP induced sepsis rats, activation of PPARγ can reduce the expression of RIPK1, RIPK3, and MLKL in myocardial tissue, reducing the secretion of pro-inflammatory cytokines, thus inhibiting myocardial necroptosis 131. A recent experiment showed that overexpression of sequestosome 2 can inhibit the formation of RIPK3-MLKL complex and necroptosis caused by MLKL translocation, thus reducing myocardial injury 123. In addition, Du et al. discovered that 35 necroptosis-related differentially expressed genes participate in sepsis-induced myocardial injury by regulating the infiltration of immune cells such as macrophages, neutrophils, and mast cells in the cardiac tissue of LPS septic mice. It is speculated that these genes may be potential therapeutic targets for sepsis-induced myocardial injury 132.

##### (3) Pyroptosis

Pyroptosis is a lytic inflammatory cell death induced by inflammation or infection, which is mainly mediated by Caspase1 (the classical pathway: activated via PRRs sensing DAMPs or PAMPs) or Caspase4/5/11 (the non-classical pathway: directly activated by pathogens) by gasdermin D (GSDMD). The activated N-terminus of GSDMD oligomerizes with phospholipids on the cell membrane, forming membrane pores. The resulting pores disrupt the osmotic pressure inside and outside the cell, leading to cell swelling, membrane dissolution, and release of cell contents (such as IL-1β and IL-18), triggering severe inflammatory responses 133. In the early stages of sepsis, appropriate pyroptosis helps to eliminate intracellular pathogens, but excessive pyroptosis can cause uncontrolled inflammatory reactions, worsening the progression of sepsis. Studies have found that knocking down the miR-21 gene can inhibit Caspase1 activation and GSDMD cleavage, thereby reducing LPS-induced pyroptosis and septic shock 134. Additionally, in a mouse sepsis model induced by CLP, zinc finger antagonistic proteins have been discovered to induce cardiomyocyte pyroptosis by regulating the AMP-activated protein kinase (AMPK)/mTOR signalling pathway, exacerbating sepsis-induced cardiac dysfunction 135. Furthermore, pyroptosis also induces cell death involved in immune responses. For example, after LPS treatment, platelets from mice with specific deletion of the GSDMD gene remain intact, while platelets from the control group exhibit swelling and membrane rupture. Further research found that mtDNA released after platelet pyroptosis induces neutrophils to produce large amounts of NETs. NET-derived S100A8/A9 further amplifies platelet pyroptosis via the TLR4-ROS-NLRP3-caspase-1 axis, establishing a self-perpetuating inflammatory cycle. S100A8/A9 can also induce platelets to release ROS and mtDNA, leading to cardiac inflammation and heart failure 136, 137.

Although pyroptosis is mainly mediated by GSDMD, recent work by Wang et al. found that LPS induces the expression of Caspase3, Caspase8, and GSDME in bone marrow macrophages derived from NLRP3 knockout mice and GSDMD knockout mice, leading to pyroptosis 138. These results suggest that pyroptosis can also be mediated by activating the expression of GSDME under conditions of high NLRP3 activation and absence of GSDMD. Similar to other members of the GSDMs family, the GSDME protein also contains an inhibitory C-terminal domain, a pore-forming N-terminal domain, and a linker region, which can be cleaved by Caspase3 to activate N-GSDME, inducing cell pyroptosis 139. Similarly, the expression of GSDMD is significantly increased in LPS-treated RAW264.7 cells, but there is no significant change in GSDME. However, siRNA-mediated GSDMD suppression induces a large amount of GSDME expression 140. This suggests that targeting the inhibition of GSDMD expression is not enough to suppress inflammation spread in sepsis models, and in future research, the GSDME-mediated pyroptosis pathway should not be ignored. In addition, studies have found that GSDME mediates cell pyroptosis in sepsis-induced liver and lung injury 141, 142. But there have been no reports related to sepsis-induced myocardial injury, representing a potential research frontier.

##### (4) Ferroptosis

Ferroptosis, first reported by Dolma et al. in cancer cells 143, is mainly caused by the insufficient redox capacity of cells and the massive accumulation of iron, leading to Fe^2+^ dependent lipid peroxidation 144. In recent years, an increasing amount of data has indicated that ferroptosis plays an important role in the pathogenesis of myocardial ischemia-reperfusion injury 145, diabetic cardiomyopathy 146, chemotherapy-induced myocardial damage, and heart failure 147. Recently, it was found that ferroptosis is also involved in sepsis-induced myocardial injury. LPS stimulation enhances the expression of nuclear receptor coactivator 4 (NCOA4) and Fe^2+^ in mouse hearts. NCOA4 promotes ferritin degradation and further releases a large amount of Fe^2+^, activating mitochondrial siderofexin (SFXN1) on the mitochondrial membrane. SFXN1 transports cytoplasmic Fe^2+^ to the mitochondria, causing mitochondrial iron overload and generating a large amount of ROS, ultimately leading to lipid peroxidation and cardiomyocyte ferroptosis 148. In addition, using ferroptosis inhibitors or iron chelators directly eliminates free iron, suppress ROS levels, block myocardial cell ferroptosis, and improve cardiac dysfunction induced by LPS in mice 149, 150.

##### (5) Autophagy

Autophagy is the cell death pathway maintaining cellular function by degrading damaged or dysfunctional cellular components 151. Research has shown that autophagy is initiated at the early stage of sepsis and plays a protective role in sepsis by increasing autophagosomes and the expression of autophagy-related proteins. In CLP-induced septic mice, the autophagy-related molecule microtubule-associated protein light chain 3 (LC3) in heart tissue peaks at 6 hours after CLP injury and gradually decreases to baseline levels at 24 hours, with the autophagic process being inhibited. However, timely administration of rapamycin (1 hour after CLP) can fully activate autophagosomes and the expression of autophagy-related molecules LC3 and Beclin1, regulate cardiac immune responses, and restore the decline in cardiac function caused by CLP 152. Therefore, in the early stage of sepsis, the immune regulatory function of the body can be improved by inducing and activating the autophagy pathway. In addition, in the LPS sepsis model, the use of resveratrol can enhance autophagic activity in mouse hearts by increasing AMPK, thus alleviating LPS-induced myocardial damage. It is manifested as enhanced the expression of LC3II and Beclin1, accumulation of autophagosomes, and reduced the level of inflammatory factors IL-1β, IL-6, and TNF-α 153. However, autophagy is a double-edged sword as excessive or insufficient autophagy under conditions of high energy consumption and nutrient deprivation can lead to the auto degradation or accumulation of toxic substances, ultimately resulting in cell death. For example, inhibiting autophagy blocks LPS-induced cardiomyocyte ferroptosis and improve cardiac function 148.

##### (6) PANoptosis

PANoptosis, a novel programmed cell death modality integrating features of pyroptosis, apoptosis, and necroptosis, was first described by Malireddi et al. in 2019. It was discovered that the innate immune sensors Z-DNA binding protein 1(ZBP1) and transforming growth factor β-activated kinase 1 (TAK1) kinase play important roles in the regulation of PANoptosis body assembly 154. In the past two years, reports have also been gradually published on the role of PANoptosis in septic lung injury 155, septic disseminated intravascular coagulation 156, and COVID-19 157. Importantly, Xiaochaihu decoction reduces LPS-induced myocardial injury in mice by inhibiting PANoptosis related gene ZBP1 158. In addition, Zhang et al. demonstrated in a CLP induced myocardial injury mice that either genetic knockdown or pharmacological inhibition of Piezo1 channels significantly attenuated myocardial injury. The protective mechanism may be associated with the regulation of calcium homeostasis and subsequent inhibition of calcium-dependent PANoptosis signalling pathway activation 159.

**Figure 3:**
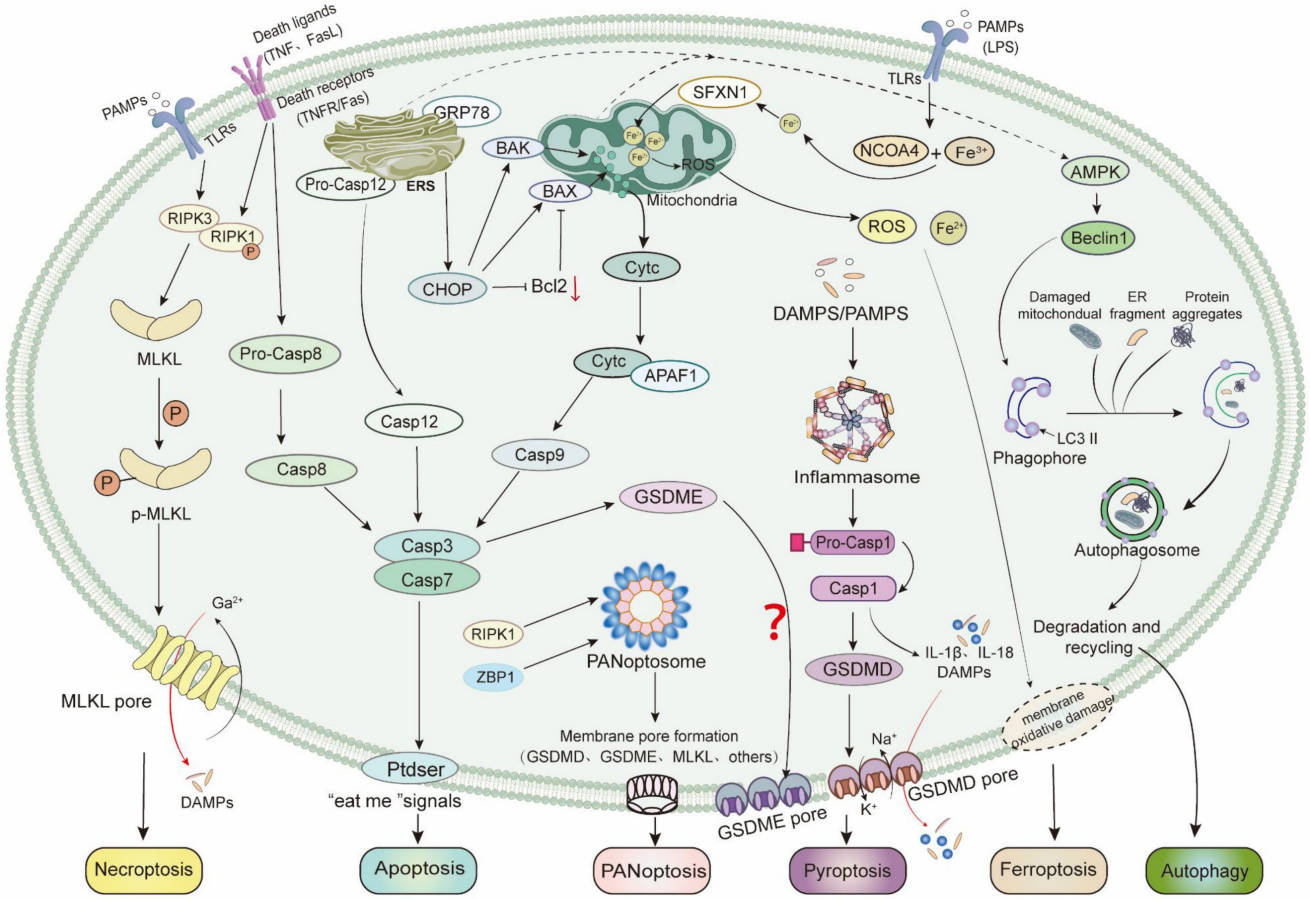
** Programmed cell death in sepsis-induced myocardial injury.** In sepsis-induced myocardial injury, there are six kinds of programmed cell death: Caspase9 (Casp9) activated by mitochondrial pathway, Casp12 activated by endoplasmic reticulum stress pathway and Casp8 activated by death receptor pathway, all of which are involved in the the process of cardiomyocyte apoptosis. RIPK1-RIPK3 forms a heterologous complex that activates MLKL, and the activated MLKL translocates to the inner and cytoplasmic membranes, initiating Ca^2+^ influx, leading to cell swelling and membrane rupture, and necroptosis. Casp1 activates GSDMD, and the activated GSDMD interacts with phosphatidylinositol on the cell membrane to oligomerization, resulting in cell membrane pores and cell apoptosis. Whether Casp3/GSDME-mediated apoptosis is involved in sepsis-induced myocardial injury has not been investigated. SFXN1-mediated ferroptosis, LC3II and Beclin1-mediated autophagy, and ZBP1-mediated PANoptosis are involved in sepsis-induced myocardial injury.

Programmed cell death is a research hotspot in sepsis. In recent decades, researchers have gained deepening insights into the cell death processes in sepsis-induced myocardial injury. The above-mentioned apoptosis, necroptosis, pyroptosis, autophagy, and the recently discovered ferroptosis and PANoptosis are all involved in the occurrence and development of sepsis-induced myocardial injury (**Figure 3**). The complex immune imbalance and the “cytokine storm” environment induce multiple death pathways. However, most current research predominantly focuses on elucidating one or two types of programmed cell death in the pathogenesis of sepsis-induced myocardial injury, with relatively single and dispersed research. The concept of PANoptosis not only provides a plausible explanation for the simultaneous changes in multiple cell death markers observed in septic myocardial injury, but more importantly, it offers researchers an integrative framework to understand the complex intracellular signalling networks and their coordinated regulation following myocardial injury. This theory has opened new research perspectives for elucidating the intricate interactions between different cell death pathways. However, the more specific molecular mechanisms of PANoptosis in sepsis-induced myocardial injury has not been elucidated, which may be a new starting point for exploring a variety of programmed death pathways in sepsis-induced myocardial injury.

### 2.3 Therapeutic application of modern medicine in sepsis-induced myocardial injury

Up to now, the definition of sepsis-induced myocardial injury has not been clearly established, and no consensus or guidelines exist for its management. However, extensive research has identified key pathophysiological features, including biventricular systolic/diastolic dysfunction, impaired responsiveness to fluid resuscitation and catecholamine stimulation, and the transient nature of injury resolution within 7-10 days. Based on these characteristics, sepsis-induced myocardial injury can be referred to as sepsis myocarditis or sepsis myocardial dysfunction 160. According to literature reviews, the following indicators can be comprehensively evaluated for diagnosis: 1) abnormal electrocardiograms, such as sinus tachycardia and atrial fibrillation 161; 2) elevated markers of myocardial injury, such as elevated cTn and brain natriuretic peptide (BNP) 162, 163; 3) abnormal echocardiographic indicators, such as lower e′ and higher E/e' 164, increased global longitudinal strain (GLS), and right ventricular wall strain 12, 165.

Due to the lack of guidelines for the management of sepsis-induced myocardial injury, the main clinical treatment strategies can be summarized as follows: 1) Vasopressors: in the conditions of septic shock, clinical treatment options include adrenaline, NA, or dopamine to increase blood pressure and restore myocardial contractility. However, a large number of studies suggest that vasopressors can increase cardiac afterload, decrease cardiac output, cause arrhythmias, and worsen cardiac function 3. Although preclinical research indicates that the weaker vasopressor, norepinephrine, can inhibit apoptosis of myocardial cells and improve cardiac function in septic rats, there is no corresponding human study 166; 2) Fluid resuscitation: patients with sepsis may have symptoms of reduced blood volume and vascular function, which correspond with the decrease in cardiac output and stroke volume. Intravenous fluid resuscitation in conjunction with vasopressors can improve the above-mentioned cardiac insufficiency. However, clinical practice has also found that fluid resuscitation may cause dilutional coagulopathy, fluid overload, and pathogenic oedema in other organs 167; 3) Positive inotropic agents: dobutamine treatment can improve cardiac function in patients with septic shock 4. However, Arnaldo pointed out that dobutamine can produce beneficial effects in patients with myocardial contractile dysfunction. However, its application is often limited by the relatively common occurrence of adverse reactions, such as left ventricular outflow tract obstruction 168; 4) β-blockers, which can reduce the oxygen demand of the myocardium, increase the filling of the heart during diastole, ultimately improving sepsis myocarditis. A retrospective study found that patients who took any β-blocker for at least one year were associated with a reduced 28-day mortality rate, providing evidence for the protective role of β-blockers in sepsis associated cardiomyopathy during sepsis treatment 169. While the above-mentioned treatment methods have certain applications in clinical practice, they all have their downsides and limited treatment effects. Therefore, researchers need to continue exploring new treatment targets and methods.

Emerging therapeutic targets for sepsis-induced myocardial injury have been identified through advancing research, demonstrating promising clinical potential. 1) NLRP3 inflammasome inhibitors: The NLRP3 inflammasome inhibitor MCC950 alleviates LPS-induced cardiomyocyte apoptosis and improves cardiac function in rats 170. Artemisinin, an anti-malarial phytochemical with immunomodulatory properties, reduces mortality, serum inflammatory cytokines, and cardiac neutrophil infiltration through NLRP3 inflammasome inhibition in burn-induced septic mice 171. Furthermore, the FDA-approved antidiarrheal drug nifuroxazide improves cardiac structure by suppressing the TLR4/NLRP3 pathway in LPS-induced cardiac inflammation 172, although NLRP3's role in sepsis-induced myocardial injury remains in early investigation. 2) Ferroptosis modulators: The wogonin from *Scutellaria baicalensi*s mitigates septic myocardial injury by regulating ferroptosis 173, while the ferroptosis inhibitor ferrostatin-1 ameliorates cardiac dysfunction and enhances survival in CLP mice 174. 3) Inflammatory cytokine modulators: Selective serotonin reuptake inhibitors elevate IL-10 levels to counteract sepsis-induced heart failure 175, IL-1 receptor antagonist shows cardioprotective potential 176, and IL-16-neutralizing antibodies improve survival and cardiac function in septic mice 177. 4) Immune cell modulators: Additionally, the immune modulator GTS-21 exerts protective effects by promoting M2 macrophage polarization, reducing oxidative stress, preserving mitochondrial function, and decreasing atrial fibrillation incidence in sepsis 178. These findings from fundamental research collectively offer novel therapeutic perspectives for sepsis-induced myocardial injury treatment.

## 3. TCM Insights into sepsis-induced myocardial injury

TCM is based on the concept of holism, with syndrome differentiation and treatment as its main feature, constituting a comprehensive system for understanding human health and disease. There is no sepsis specifically recorded in TCM. According to Chinese medical theory, sepsis belongs to categories such as “Cold Damage”, “Exogenous Febrile Diseases”, and “Warm Toxins”, and can be considered a result of the invasion of “Blood Stasis” and “Pathogenic Toxin” in the body. “Blood stasis” refers to impaired microcirculation, leading to local or systemic blood stasis. “Pathogenic Toxin” encompasses external pathogens or endogenous toxins that harm the body. When pathogenic toxins invade the body, especially when spread through the blood, they can cause systemic inflammatory reactions, accumulate in the organs, and endanger life.

### 3.1 Etiology and pathogenesis of sepsis

Sepsis is a multifactorial clinical syndrome. Although modern TCM scholars have different views on its etiology and pathogenesis, the causes of sepsis can be generally divided into exogenous toxins and endogenous toxins 179. Exogenous toxins include the six pathogenic factors (wind, cold, heat, dampness, dryness, and fire), and direct or indirect contact with toxic substances such as external injuries, poisonous food, allergens, and pestilential Qi. The toxins travel along the meridians, directly attack the organs, and invade the body. Endogenous toxins include the Qi-blood disharmony and visceral dysfunction. If the pathogenic factors cannot be expelled from the body in time, stasis, phlegm, heat, etc., may form over time. Additionally, special consideration should be given to the patient's constitution when it comes to endogenous toxins of sepsis. When the nature of the external pathogen differs from the constitution, such as a body with excessive Yang Qi encountering a cold pathogen, it may transform into a heat pathogen, or a body with excessive Yin encountering a hot pathogen, it may transform into dryness. These all show that the external pathogens change with the constitution, manifesting the phenomenon of “differential manifestations from identical etiology”. When the pathogen is similar to the constitution, the occurrence pattern of the disease depends on the waxing and waning of the pathogenic factors and the Zheng Qi (healthy energy). In conclusion, the internal disharmony of Qi and blood, visceral dysfunction, deficiency of Zheng Qi, and exuberance of external pathogenic factors intertwine, leading to Qi stagnation, impairing the collaterals and viscera, and ultimately resulting in the occurrence of sepsis.

In the Ming Dynasty, the famous physician Zhang Jiebin proposed: “The mechanism is the essence and the transformation, and the disease is caused by the change”. The concept of disease pathogenesis describes the occurrence and development of disease, which is the basis of TCM in diagnosis and treatment. The core pathogenesis of sepsis is “deficiency, heat, toxin, and stasis” 180.

Firstly, the *The Yellow Emperor's Inner Canon (Huangdi Neijing)* states: “When the Zheng Qi is abundant, pathogens cannot invade”. Chinese medicine believes that Zheng Qi is the body's defensive matrix. If the Zheng Qi is strong, even if invaded by pathogens, it can fight against them. Therefore, many medical experts also believe that “impairment of Zheng Qi” is the fundamental mechanism of sepsis development 181. Secondly, sepsis belongs to the categories of “externally contracted febrile disease” and “warm syndrome”. Internal and external toxins disturb the body, cause internal heat, generate internal toxins, form symptoms of internal heat bias, damage organs, and deplete vitality. Thirdly, based on the theory of “heat syndrome”, Professor Zhu Hai and others have developed the theory of “toxins”. The internal presence of toxins is the basis for the onset of sepsis. The internal generation of turbid toxins leads to poisoning internally, stagnation of triple energizer water and Qi, organs suffering from fire burning, dysfunctional organs, and the development of various organs' reversal symptoms. Finally, the formation of blood stasis is closely related to the development of sepsis. If the blood is deficient, stasis will form, or the heat toxin forces the blood to overflow, causing stasis. Conversely, blood stasis will worsen the condition of sepsis, causing new damage to organs and systems.

In summary, sepsis patients suffer from deficiency of Zheng Qi, invasion of external pathogenic factors, and inability to expel the evils, leading to external pathogenic factors. As the disease progresses, the struggle between pathogenic factors and the Zheng Qi, blood, and body fluids, leads to stasis. The accumulation of toxins in the body causes disharmony in Yin and Yang, Qi and blood, body fluids, and visceral function, ultimately exacerbating the deficiency pattern 182. These four pathogenic elements—deficiency, heat, toxin, and stasis—coexist synergistically, dominating distinct pathophysiological stages of sepsis progression.

### 3.2 TCM pathogenesis of sepsis-induced myocardial injury

The heart is located in the chest, between the two lungs, and is surrounded by the pericardium for protection. The heart governs the blood vessels, propelling the circulation of blood, nourishing the organs, and moistening the entire body. The heart also houses the spirit, overseeing the physiological activities of the entire body's organs, meridians, acupoints, and physical form, as well as coordinating mental changes such as thoughts, consciousness, spirit, and emotions. Therefore, the heart is referred to as the “monarch organ” in TCM theory.

The systemic inflammatory response caused by sepsis, particularly toxins and stagnant blood in the blood, disrupts the circulation of Qi and blood in the body, leading to Qi stagnation and blood stasis, as well as heat-toxin accumulation. The heart is the ruler of the Five Zang and Six Fu Organs. When the lungs are invaded by pathogens, it affects the heart's blood circulation. Kidney failure leads to water and Qi counterflow affecting the heart and lungs. The liver failing to store blood results in the accumulation of toxins over time, illness of mother viscera affecting the child one. Spleen deficiency causes phlegm production internally illness of child viscera affecting the mother one, all of which can lead to heat and toxicity stagnating in the heart, affecting its normal function. Conversely, if pathogenic toxins reach the heart, the heart will be too weak to propel blood circulation, leading to another blockage in the flow of Qi and blood, and failing to nourish the other organs and tissues.

#### 3.2.1 Disease pattern

TCM does not have specific records of sepsis-induced myocardial injury. However, based on its clinical symptoms and progression, it shares many similarities with the warm febrile diseases discussed in *Treatise on Cold Damage (Shang Han Lun)* and *Systematic Differentiation of Warm Diseases (Wen Bing Tiao Bian)*. It is often attributed to the invasion of external pathogenic toxins. Currently, sepsis is classified into several TCM syndrome patterns, including toxin-blood syndrome, blood stasis syndrome, Fu-organ Qi obstruction, and acute deficiency syndrome. The treatment principles mainly include “clearing heat and detoxifying”, “activating blood circulation and resolving stasis”, “unblocking and descending”, and “strengthening Zheng Qi”.

Furthermore, as stated in *Basic Questions (SuWen)*, “All heat diseases are categories of cold damage”. Sepsis can be categorized under “external contraction heat diseases”, “warm diseases”, “syncope syndrome”, and “collapse syndrome”. Sepsis-induced myocardial injury can be classified under TCM disease patterns such as “palpitations”, “chest impediment”, “dyspnea”, and “edema”. Common clinical manifestations include palpitations with insomnia, Qi-blood stagnation, and heart Yang insufficiency.

#### 3.2.2 Yin-Yang theory

There is no record of myocardial injury in TCM. From the perspective of TCM, modern scholars classify it as “chest obstruction” or “oedema” based on the clinical manifestations of patients. Common symptoms include palpitations, insomnia, Qi stagnation, blood stasis, and deficient heart Yang. Professor Sun Qi and his team summarized and analyzed the pattern of changes in septic cardiomyopathy from the perspective of Yin-Yang theory. In patients with Yang Qi deficiency, pro-BNP levels are highest, stroke volume, speed index of myocardial contraction, and acceleration index decrease, and peripheral vascular resistance increases, indicating deficient heart Yang, weak pumping force, blood stasis, and impaired circulation 183.

#### 3.2.3 Triple energizer theory

Professor Kong Li, based on the theory of the triple energizer, believes that addressing the upper energizer to restore the functions of the heart and lungs is crucial in treating septic cardiorenal syndrome. This can improve heart function, reduce burden, tonify heart Qi, strengthen heart Yang, restore the flow of Qi in the lungs, assist in blood circulation, and advocate for the supplementation of heart Yang 184.

#### 3.2.4 State target medicine theory

Academician Tong Xiaolin proposed the modernization of TCM thinking through the “State Target Medicine” theory, aiming to achieve comprehensive disease management and precise treatment 185. The “State” refers to the condition, dynamics, and trend of the internal environment of the human body, providing an overall summary of the stage-specific characteristics of a disease and extracting the core pathogenesis at each stage. The “Target” includes the patient's definitive disease diagnosis (disease target), abnormal symptoms and signs (symptom target), and abnormal indicators (marker target), serving as a critical tool for achieving precision medicine. The three states of “toxin, “stasis”, and “deficiency” can comprehensively summarize the clinical manifestations of sepsis-induced myocardial injury.

In the “toxicity state”, the patient's body has not yet experienced deficiency. At this stage, the selection of herbal medicine should focus on clearing heat and detoxifying, such as *Rhubarb palmatum* and *Houttuynia cordata*. In the “stasis state”, patients often present with concurrent issues such as coagulation dysfunction, organ ischemia, or thrombosis. The selection criteria for herbal medicine at this stage should emphasize activating blood circulation, removing stasis, cooling blood, and reducing swelling. For example, tanshinone IIA, an active component of *Salvia miltiorrhiza*, has been shown to inhibit myocardial cell autophagy in septic mice and improve cardiac function 186. In the “eficiency state” Shenfu Injection can be utilized. Shenfu Injection has been demonstrated to enhance myocardial systolic and diastolic functions, effectively alleviating myocardial injury in septic patients 187.

#### 3.2.5 Four Syndromes and Four Methods

As clinical research on sepsis progresses, the China Consensus on Early Prevention and Emergency Blockage of Sepsis in 2020 suggests using the “our Syndromes and Four Methods”to diagnose and treat sepsis. That is, toxic-heat syndrome, blood stasis syndrome, acute deficiency syndrome, and visceral Qi dysfunction syndrome, which also apply to septic cardiomyopathy. A recent clinical trial has shown that using the “our Methods”significantly restores levels of cTnI and pro-BNP in sepsis patients, reduce levels of inflammatory factors such as IL-6 and IL-10, and improve heart function. The single diagnostic method has no statistical significance, which also reflects the complexity of sepsis-induced myocardial injury mechanism 188.

### 3.3 Therapeutic application of TCM in sepsis-induced myocardial injury

The myocardial damage caused by sepsis is often classified as “hest obstruction”or “oedema” in TCM. For the treatment of myocardial damage, TCM mainly uses methods such as clearing heat and detoxification, promoting blood circulation and resolving stasis, strengthen the root and secure the foundation (Fuzheng Guben), dredge the Fu-organs and promote purgation, tonifying Qi and nurturing the heart. It can be used as an adjuvant therapy in modern medicine to improve clinical efficacy and patient prognosis. Yang and his colleagues treated the patient for 5 days with the heat-clearing and detoxifying method with Po-Ge-Jiu-Xin decoction. The C-reactive protein, cTnl, pro-BNP, and white blood cell count of the patients are significantly decreased, while LVEF value is higher, effectively improving the heart function of sepsis patients 189. Additionally, the modified detoxifying Shengmai San developed based on Shengmai San and Qingying Tang, can Fuzheng Guben and increase LVEF level, CD4^+^T and CD8^+^T lymphocyte levels, inhibit BNP level and white blood cell count, to significantly improve the inflammatory response of sepsis patients, enhance immune function, and improve heart function 190. A clinical trial found that Jinhong Tang can significantly reduce SOFA score, cTnl, pro-BNP levels, TNF-α, IL-6, and other inflammatory factors levels, improve prognosis, and reduce organ failure risk 191. Currently, a large number of Chinese herbal compound formulas have been proven to have good effects in treating sepsis and its myocardial damage. In addition to the above examples, Xuebijing injection (promote blood circulation and removes blood stasis), Shenfu injection (dredge the Fu-organs and promote purgation), and other Chinese patent medicines have been widely used in clinical practice, achieving certain therapeutic effects. These compound formulas and Chinese patent medicines have multiple ingredients, and comprehensive anti-inflammatory, antibacterial, and antioxidant effects, providing comprehensive regulatory effects on sepsis and myocardial damage.

TCM compound formulations demonstrate unique therapeutic advantages in the treatment of sepsis-induced myocardial injury. Taking Xuebijing Injection as an example, this formulation is scientifically composed of multiple herbal medicines including *Chuanxiong Rhizoma*, *Radix Paeoniae Rubra*, *Salvia miltiorrhiza*, and *Carthamus tinctorius*. Its multi-component and multi-target characteristics enable comprehensive regulation of pathological states through synergistic effects. A randomized controlled trial demonstrated that sepsis patients treated with Xuebijing injection showed significant improvement in cardiac function indicators such as LVEF compared to the control group 192. In basic research, Wang et al. identified paeoniflorin and safflor yellow A as the primary active compounds in Xuebijing responsible for improving cardiac function via RNA-seq analysis. The protective mechanism was mediated by suppressing the expression of IL-6, IL-1β, and CXCL2 67. Additionally, studies have revealed that Xuebijing exerts cardioprotective effects by inhibiting TLR4/IKKα-mediated NF-κB and JAK2/STAT3 signaling pathways, thereby attenuating inflammatory responses and apoptosis during sepsis 193. Shenfu injection, a well-known classical Chinese patent medicine for sepsis treatment, was investigated by Huang et al. using network pharmacology. Their study revealed that Shenfu injection suppresses inflammatory responses by inhibiting IL-2 and TNFα-related molecules, while also enhancing anti-apoptotic effects through upregulation of Bcl2 and downregulation of Caspase-9. These mechanisms collectively improve survival rates and cardiac function in septic mice 194.

However, the chemical complexity of compound formulations also presents challenges for mechanistic studies, quality control, and standardization. With advancements in modern separation and purification technologies, coupled with progress in pharmacological research methodologies, significant breakthroughs have been achieved in the screening of active TCM components. Systematic screening and identification of effective components not only elucidate the material basis of TCM's therapeutic effects but also provide scientific evidence for understanding its molecular mechanisms in disease prevention and treatment. Notably, TCM extracts and their active components, characterized by unique chemical structures and well-defined molecular targets, demonstrate significant advantages in therapeutic predictability and quality control. These advancements have established a crucial foundation for the modernization and internationalization of TCM.

Ginsenoside Rg1, one of the primary active constituents of *Panax ginseng*, has demonstrated significant therapeutic effects in anti-inflammatory diseases. Utilizing both CLP-induced sepsis myocardial injury models and LPS-induced cardiomyocyte sepsis models, Liu et al. demonstrated that Ginsenoside Rg1 could suppress the pro-apoptotic protein Bax, activate Akt, induce GSK-3β phosphorylation, inhibit apoptosis, and restore mitochondrial calcium homeostasis in cardiomyocytes, thereby ameliorates cardiac function in septic mice 195. Berberine, a prominent isoquinoline alkaloid with significant bioactivity found in *Berberis* species, has shown promising therapeutic potential. Recent research by Shen et al. revealed that berberine protects mitochondrial function and increase ATP content in cardiomyocytes and exerts anti-inflammatory effects through activation of the Notch signaling pathway in CLP-induced septic rats, thereby attenuating myocardial injury and improving cardiac function 196. Furthermore, puerarin, an isoflavone compound extracted from *Pueraria lobata*, exhibits remarkable cardioprotective effects. Zhou et al. demonstrated that puerarin inhibited AMPK phosphorylation and decreased the expression of IL-6 and TNFα in LPS-induced myocardial injury in rats, thereby reducing myocardial tissue inflammation and oxidative damage, inhibiting myocardial cell apoptosis and ferroptosis, and finally exerting myocardial protection 197.

Extensive research has identified numerous bioactive components derived from TCM that exhibit significant protective effects against sepsis-induced myocardial injury (**Table 2**). The underlying mechanisms involve multiple pathways, including anti-inflammatory actions, antioxidant effects, anti-apoptotic properties, and inhibition of ferroptosis. These multi-target and multi-pathway mechanisms provide a crucial theoretical foundation for the prevention and treatment of sepsis-induced myocardial injury using TCM.

In addition, acupuncture, tuina, and other treatments are also applied in the treatment of sepsis and its myocardial injury. Acupuncture can regulate the body's Qi and blood circulation, promoting the elimination of pathogenic factors. For example, electroacupuncture at the “Neiguan” acupoint inhibits inflammatory responses through vagus nerve mediated cholinergic pathways, thereby alleviating left ventricular dysfunction in septic rats 198. Electroacupuncture at “Zusanli” can significantly reduce the levels of pro-inflammatory factors at 6h after CLP, and play a protective role in the heart 199. Tuina therapy can improve Qi and blood circulation, promoting the recovery of the condition. In recent years, traditional Chinese tuina therapy has played a good role in the prevention and treatment of cardiovascular and respiratory-related diseases such as coronary heart disease, heart failure, arrhythmias, and chronic obstructive pulmonary disease 200. In some clinical experiments, after giving basic treatment to patients with sepsis-related malnutrition after mechanical ventilation, the experimental group underwent acupoint tuina and meridian tuina on top of the basic treatment. It was found that after the intervention, the nutritional tolerance score of the patients was significantly lower than that of the control group and the acupoint tuina group. It is suggested that acupoint tuina along meridians is beneficial to prevent enteral nutrition intolerance in patients with sepsis 201. These therapies are often used in combination with drug treatments, achieving good therapeutic effects. Overall, TCM has unique advantages and rich experience in the treatment of sepsis and its associated myocardial injury, but further clinical and basic research is still needed to verify its efficacy and mechanism.

## 4. Discussion and Conclusion

### 4.1 Discussion on pathogenesis in modern medicine and TCM

Modern medicine posits that the molecular mechanisms of sepsis-induced myocardial injury involve immune dysregulation, cytokine storms, oxidative stress, mitochondrial dysfunction, coagulation abnormalities, and ultimately, excessive cell death leading to cardiac dysfunction. These processes are highly complex and interconnected. In contrast, TCM does not have specific concepts such as “sepsis” or “sepsis-induced myocardial injury”. Instead, TCM views the progression of disease as a dynamic and evolving process rather than a static condition. Based on clinical symptoms and disease progression, sepsis-induced myocardial injury can be categorized under TCM syndromes such as “palpitations”, “chest obstruction”, “panting syndrome”, and “edema”.

In the initial phase of sepsis, TCM theory holds that Zheng Qi deficiency and invasion of pathogenic factors lead to Qi-blood disharmony, impairing the body's ability to expel pathogenic influences in a timely manner. Over time, these factors transform into heat, manifesting clinically as heat-toxin accumulation, thereby presenting as the “heat-toxicity syndrome”. From a modern medical perspective, the immune dysregulation in sepsis and the resulting cytokine storm are often accompanied by various thermoregulatory responses, which align with TCM observations. In this context, Zheng Qi can be interpreted as the body's immune competence. When the body is invaded by pathogens or pathogenic factors, this defense mechanism is rapidly activated. Through PRRs, the immune system identifies threats and mobilizes adaptive immune cells to eliminate pathogens. However, excessive immune cell activation can lead to immune dysregulation, ultimately contributing to cardiac impairment.

As the disease progresses, heat-toxin accumulation damages vasculature, causing reckless blood flow and stasis formation, manifesting as a “blood stasis syndrome”. Modern medicine correlates this with endothelial injury and microcirculatory dysfunction, resulting in disseminated intravascular coagulation. “Blood stasis syndrome” can be interpreted as coagulation dysfunction. During sepsis pathogenesis, excessive activation of immune cells and inflammatory factors, particularly NETs, triggers the coagulation cascade. The nucleic acid components within NETs activate factor VII to VIIa, subsequently generating factor Xa. Factor Xa, together with factor V, converts prothrombin to thrombin, thereby promoting fibrin formation. Furthermore, histones in NETs activate platelets, further amplifying the coagulation pathway and inducing microthrombus formation. The overproduction of thrombin and fibrin leads to microangiopathy, which may progress to DIC, exacerbating multiple organ dysfunction (including cardiac impairment) and increasing mortality risk.

In the advanced phase, the body's Zheng Qi becomes severely deficient, impairing its ability to promote blood circulation. This leads to blood stasis and obstruction of the vessels, manifesting as a “deficiency syndrome”. From a modern medical perspective, “deficiency syndrome” can be understood as “heart failure”, this corresponds to the cessation of microcirculatory blood flow, characterized by a lack of perfusion and flow. Tissues are deprived of adequate oxygen and nutrients, and the microvasculature becomes dilated and unresponsive to vasoactive agents, indicating microcirculatory failure. Furthermore, during sepsis pathogenesis, the inflammatory response rapidly activates the sympathetic nervous system. Under the influence of the hypothalamic-pituitary-adrenal axis, this enhances the release of catecholamines such as epinephrine from postganglionic sympathetic neurons and the adrenal medulla. The cardiovascular system, being rich in adrenergic receptors, is particularly susceptible to excessive stimulation from sympathetic activation. This leads to apoptosis of preganglionic neurons and glial cells in cardiac autonomic centers, impaired systolic and diastolic function, tachyarrhythmias, decreased cardiac sensitivity to intrinsic catecholamines, and compromised autonomic control of both heart and blood vessels.

In summary, while modern medicine elucidates the molecular mechanisms of sepsis-induced myocardial injury through a detailed biological lens, TCM interprets the condition as a dynamic process of syndrome evolution, emphasizing the interplay between heat, stasis, and deficiency. Integrating these perspectives may provide a more comprehensive understanding and therapeutic approach to managing sepsis-induced myocardial injury.

### 4.2 Discussion on diagnosis in modern medicine and TCM

Modern medicine employs various diagnostic methods for sepsis-induced myocardial injury, including echocardiography, electrocardiography, hemodynamic monitoring, and biomarkers of myocardial injury (such as cTn and BNP). Despite significant advancements in understanding the mechanisms and diagnostic approaches for sepsis-induced myocardial injury, there remains a lack of authoritative diagnostic guidelines. In contrast, TCM is guided by the principle of "holism" and characterized by “syndrome differentiation and treatment”. It emphasizes the harmonious relationship between humans and their natural environment, aiming to address both localized and systemic disorders by restoring the body's internal equilibrium. TCM focuses on the dynamic interactions among “disease, the human body, and the environment”, offering a personalized diagnostic and therapeutic strategy that serves as a valuable complement to modern medicine.

Notably, modern medical research has shifted from a narrow focus on eradicating pathogenic microorganisms to modulating the body's immune responses. Contemporary medicine now underscores the intrinsic connections among systemic organs, as evidenced by theories such as hepatorenal syndrome, cardiorenal syndrome, and the gut-lung axis. This aligns with TCM's holistic concept of dynamically regulating multiple systems within the patient 202.

Furthermore, tongue diagnosis is a unique diagnostic method in TCM, with ancient texts stating, “In chronic conditions, follow the pulse; in acute conditions, examine the tongue”. This highlights the critical role of tongue diagnosis in the management of acute and critical illnesses. A clinical retrospective study demonstrated that combining the SOFA score, BNP levels, and abnormalities in sublingual collaterals can provide a comprehensive diagnostic approach for sepsis-induced myocardial injury. Therefore, integrating the strengths of both modern medicine and TCM can enhance the assessment of disease progression in patients with sepsis-induced myocardial injury, offering a more holistic and effective clinical strategy.

### 4.3 Discussion on therapeutic approaches in modern medicine and TCM

Currently, there is no unified management guideline for the treatment of sepsis-induced myocardial injury. However, the therapeutic approaches can be summarized as follows: infection control, fluid resuscitation, circulatory support, vasoactive agents, and inotropic drugs. While these methods are clinically applied to varying extents, each has its advantages and limitations. Based on the etiology and pathogenesis, the mainstream TCM approach to sepsis can be categorized into the “Four Syndromes and Four Methods”, which is also applicable to sepsis-induced myocardial injury. For instance, following the principle of “clearing heat and detoxifying”, Huanglian Jiedu Decoction is used to treat syndromes characterized by excessive heat-toxicity in the triple energizer. Its mechanism is associated with reducing inflammatory responses, aligning with modern medical principles. Similarly, under the principle of “promoting blood circulation and resolving stasis”, Qishen Huoxue Granules are employed to invigorate blood circulation, resolve stasis, and tonify Qi. Its mechanism is linked to improving mitochondrial dysfunction in cardiomyocytes and mitigating oxidative stress, which corresponds to modern medical understanding. In summary, while conventional treatments for sepsis-induced myocardial injury focus on infection control and hemodynamic stabilization, TCM offers complementary strategies rooted in syndrome differentiation and holistic regulation. The integration of these approaches may provide a more comprehensive therapeutic framework for managing this complex condition.

TCM demonstrates certain advantages in the treatment of sepsis-induced cardiomyopathy, providing new insights and theoretical foundations for future integrative approaches combining TCM and modern medicine. For example, studies have found that the combined use of metoprolol and Shenmai Injection in patients with septic shock can effectively suppress serum levels of CK-MB, CPK, and LDH, improve hemodynamic stability, attenuate systemic inflammatory responses, and reduce myocardial injury. Shenmai Injection is a TCM preparation derived from red ginseng and ophiopogon japonicus. In TCM theory, red ginseng reinforces healthy Qi and consolidates bodily resistance, while ophiopogon root nourishes Yin and promotes fluid production. Metoprolol, a highly selective β-adrenergic receptor blocker, is commonly used in the treatment of myocardial disorders. It inhibits β-receptor activity, thereby reducing myocardial contractility, promoting diastolic function, and improving coronary blood supply. Although metoprolol carries the risk of bradycardia, its combination with Shenfu Injection demonstrates favorable safety by modulating heart rate and preventing excessive reduction that could lead to arrhythmias, indicating a synergistic cardioprotective effect 203. Additionally, Xuebijing Injection, known for its heat-clearing, detoxifying, and blood-activating effects, combined with the antibiotic meropenem, significantly suppresses the expression of BNP, cTnI, and PCT in sepsis patients, further enhancing clinical efficacy. Meropenem, a broad-spectrum antibacterial agent, effectively kills or inhibits bacterial growth, reducing infection-induced stimulation of the coagulation system. Meanwhile, Xuebijing's active components (safflower and peony root) exert potent anti-thrombotic and microcirculation-improving effects. When used together, they may synergistically target both the coagulation system and infectious foci, thereby ameliorating microcirculatory dysfunction and improving sepsis prognosis 204.

In summary, TCM formulations that reinforce Zheng Qi can counterbalance the heart rate-lowering effects of β-blockers, while blood-activating and stasis-resolving TCM agents can synergize with antibiotics to enhance antimicrobial and microcirculatory modulation. This demonstrates that integrative therapy combining TCM and modern medicine can complement each others strengths, offering a more comprehensive approach to treating sepsis-induced myocardial injury. However, much of the current research on the effects of herbal interventions for sepsis-induced cardiomyopathy remains confined to basic studies. To obtain higher-quality evidence-based medical data, large-scale randomized controlled trials on TCM formulations for sepsis-induced cardiomyopathy are urgently needed. Additionally, there is a lack of sufficient clinical data and diagnostic and therapeutic experience in this field within China. Furthermore, the unique complexities of sepsis-induced cardiomyopathy itself necessitate extensive clinical trials for further in-depth investigation.

Moreover, in the process of modernizing TCM, the standardization of disease nomenclature has emerged as a primary challenge in integrating TCM with modern medicine. Diseases diagnosed under modern medical frameworks may appear similar to traditional TCM disease names, but they are fundamentally different. A simplistic alignment of the two can lead to significant clinical errors. For example, “chest obstruction”, a TCM condition characterized primarily by chest pain, has been inappropriately equated by some practitioners with coronary heart disease in modern medicine. Directly applying ancient TCM syndrome patterns related to “chest obstruction” to diagnose and treat coronary heart disease patients is a misalignment that can result in improper management.

In the future, it is essential not only to leverage modern technologies to further investigate the mechanisms of TCM interventions for sepsis-induced cardiomyopathy but also to focus on how to more effectively integrate TCM with modern medicine through syndrome differentiation and treatment. This integration represents a critical direction for continued attention and efforts, aiming to optimize therapeutic strategies for sepsis-induced cardiomyopathy.

## 5. Conclusion

The myocardial injury of septic cardiomyopathy is one of the main reasons for the high mortality in patients with sepsis. Exploring the pathogenesis of septic cardiomyopathy is crucial for finding new treatment methods. This review summarizes the pathogenesis and treatment strategies of sepsis-induced myocardial injury from the perspective of modern medicine, including immune dysfunction, cytokine storm, oxidative stress, mitochondrial dysfunction, abnormal adrenergic pathway, and ultimately excessive programmed cell death. At the same time, it also elucidates the etiology and pathogenesis of sepsis-induced myocardial injury from the perspective of TCM. Based on the patterns of “toxin-heat syndrome”, “blood stasis syndrome”, “acute deficiency syndrome”, and “intestinal Qi stagnation syndrome”, a treatment strategy known as the “Four Syndromes and Four Methods” is proposed, which includes “clearing heat and detoxifying”, “promoting blood circulation and resolving stasis”, “Fuzheng Guben”, and “dredge the Fu-organs and promote purgation”. Additionally, sepsis-induced cardiomyopathy is categorized under TCM syndromes such as “palpitations”, “chest obstruction”, “panting syndrome” and “edema”. Furthermore, the application of acupuncture, tuina and other TCM therapies in patients with sepsis-induced myocardial injury is combed. The multi-pathway and multi-target therapeutic advantages and the unique system of differentiation and treatment in Chinese medicine can complement the shortcomings of modern basic medical research focusing on single targets or pathways. Advanced detection methods, technologies, and instruments in modern medicine provide a theoretical basis and material foundation for the development of TCM. Therefore, in future research, we should not only consider treating the “cause” or the “effect” singly but should pay more attention to “treating both symptoms and root causes” and “individualized treatment”. We should fully tap into the potential of TCM, leverage the advantages of modern medicine in detection, and achieve complementary advantages of integrated Chinese and modern medicine treatment, providing a diversified and effective treatment plan for sepsis-induced myocardial injury.

## Figures and Tables

**Table 1:** Common cytokines and their functions in sepsis-induced myocardial injury.

Cytokines	Main cell source	Target cells	Clinical effect	Detection methods	Modeling methods	Main functions	References
IL-1β	Macrophages, epithelial cells	Cardiomyocytes	Cardiodepressant inflammatory cytokines	qRT-PCR	CLP/LPS	Promote inflammation	48
IL-6	T cells, macrophages, endothelial cells	Macrophages, neutrophils, monocytes, NK cells, T cells	Prognostic marker	qRT-PCR; Administer exogenous IL-6	CLP/LPS	Promote inflammation; promote endoplasmic reticulum stress, promote mitochondrial dysfunction, antioxidant stress	52, 55
IL-12	Macrophages, neutrophils, dendritic cells	Monocytes, NK cells	IL-12p40 protects septic myocardial injury; IL-12p35 aggravates septic myocardial injury	qRT-PCR; western blot	LPS/CLP	Anti-inflammatory, promote inflammation	56, 57
IL-17	T cells, NK cells	Endothelial cells, macrophages	pro-inflammatory cytokines	qRT-PCR; ELISA Kit	LPS	Promote inflammation	205
IL-18	Macrophages, epithelial cells	T cells, NK cells	Cardiodepressant inflammatory cytokines	qRT-PCR; western blot	LPS	Promote inflammation	49
IL-27	Macrophages, dendritic cells	T cells, IL-27RA	Anti-inflammatory cytokines	ELISA Kit	LPS	Anti-inflammatory	206
IL-35	T cells, B cells	T cells	Anti-inflammatory cytokines	qRT-PCR; western blot	LPS	Anti-inflammatory	207
CCL2/ MCP1	Macrophages, dendritic cells, cardiomyocytes	Monocytes	Pro-inflammatory cytokines	ELISA Kit	CLP/LPS	Promote inflammation	62
CXCL8/IL-8	Monocytes, macrophages, neutrophils, endothelial cells	Neutrophils	Pro-inflammatory cytokines	ELISA Kit	CLP/LPS	Promote inflammation	62
CXCL2/MIP2	Monocytes, neutrophils	Neutrophils	Recruit neutrophils	Administer exogenous CXCL2	CLP/LPS	Recruitment granulocytes, promote apoptosis	66
G-CSF	Monocytes, macrophages	Neutrophils	Recruit granulocytes	ELISA Kit	LPS	Promote inflammation	69
TNF-α	Multiple kinds of cells	Multiple kinds of cells	Cardiac function predictor	Administer exogenous TNF-α	CLP/LPS	Inhibit intracellular Ca^2+^ concentration, promote inflammation	72, 208

**Table 2:** ** Therapeutic effect of TCM on sepsis-induced myocardial injury.** PINK1, Parkin mediated mitophagy 1; JAK2, Janus kinase 2; p-AKT1, Phosphorylated serine/threonine kinase 1; PCT, procalcitonin; hs-CRP, hypersensitive C-reactive protein; xCT, cystine/glutamate antiporter; GPX4, glutathione peroxidase 4; SIRT1, sirtuin 1; DRP1: dynamin-related protein 1.

Name	Function	Validation model	Administration	Effect	Reference
Po-Ge-Jiu-Xin decoction	Clearing heat and detoxification (Qingre Jiedu)	CLP-induced Sprague-Dawley (SD) rats	25.83, 51.66 g/kg	Activation of PINK1/ parkin mediated mitophagy maintains mitochondrial homeostasis	209
Huanglian Jiedu decoction	Clearing heat and detoxification (Qingre Jiedu)	CLP-induced Balb/c mice	3.5 g/kg	Reduce inflammatory factors such as TNF-α and IL-6 to play an anti-inflammatory role	210
Xuebijing injection	Promoting blood circulation (Huoxue Huayu)	CLP-induced SD rats	40 mg/kg	Inhibition of NF-κB and JAK2/STAT3 pathway exerted anti-apoptosis and anti-inflammation	193
Dtoxifying Shengmai San	Fuzheng Guben	Patients with sepsis	Not Applicable	Suppress inflammation and improve immune function	190
Shenfu injection	Fuzheng Guben	CLP-induced SD rats	10 mL/kg	Inhibition of STAT3 and p-AKT1 exerted anti-inflammation and anti-apoptosis	187
Jinhong Decoction	dredge the Fu-organs and promote purgation	Patients with sepsis	Not Applicable	Reduce the levels of cTnI, BNP, PCT and hs-CRP in patients with early sepsis	211
YiQiFuMai injection	Qi-tonifying and Blood-activating (Yiqi Huoxue)	CLP-induced SD rats	0.23, 0.45, and 0.9 mg/kg	Enhance the xCT/GPX4 axis to inhibit ferroptosis	212
Ginsenoside-Rg1	Anti-inflammatory, anti-oxidative	CLP-induced Balb/c mice	35, 70 mg/kg	Enhance the expression of Bax and activate the Akt to inhibit apoptosis	195
Berberine	Anti-inflammatory, anti-oxidative	CLP-induced SD rats	50 mg/kg	Regulation of Notch1 signaling pathway protects myocardial mitochondria	196
Puerarin	Anti-inflammatory, anti-oxidative, anti-apoptosis	LPS-induced SD rats	100 mg/kg	Inhibition of AMPK-mediated ferroptosis	197
Tanshinone IIA	Anti-inflammatory, anti-oxidative, anti-apoptosis	LPS-induced Balb/c mice	5, 10, 50 mg/kg	Promote autophagy, inhibit NLRP3 Inflammasome	186
Emodin	Anti-inflammatory, anti-oxidative	LPS-induced C57BL/6 mice	20 mg/kg	Inhibition of NLRP3 inflammasome reduces inflammation and pyroptosis	213
Curcumin	Anti-inflammatory, anti-oxidative	LPS-induced C57BL/6 mice	80 mg/kg	Activation of SIRT1/DRP1/PGC-1α promotes mitochondrial biogenesis	214
Resveratrol	Anti-inflammatory, anti-oxidative	CLP-induced SD rats	10, 30, 50 mg/kg	Activation of Sirt1/Nrf2 signaling pathway inhibits ferroptosis	215

## Abbreviations

AR: adrenergic receptor
ATP: adenosine triphosphate
BNP: brain natriuretic peptide
CCL: CC chemokine ligand
CK-MB: creatine kinase isoenzyme MB
CLP: cecal ligation and puncture
CO: cardiac output
CSF: colony stimulating factor
cTnI: cardiac troponin I
DAMPs: damage-associated molecular patterns
DRP1: dynamin-related protein 1
EF: ejection fraction
ER: endoplasmic reticulum
ETC: electron transport chain
FS: fractional shortening
G-CSF: granulocyte
GLS: global longitudinal strain
GSDMD: gasdermin D
GSH: glutathione
GSH-Px: glutathione peroxidase
GRP78: glucose regulator protein 78
ICU: intensive care unit
iNOS: inducible nitric oxide synthase
IL: interleukin
LC3: light chain 3
MCP-1: monocyte chemo-attractant protein 1
MIP2: macrophage inflammatory protein 2
MLKL: mixed-lineage kinase domain-like
mPTP: mitochondrial permeability transition pore
MODS: multiple organ dysfunction syndrome
mtDNA: mitochondrial DNA
NA: noradrenaline
NETs: neutrophil extracellular traps
NF-κB: nuclear factor kappa B
NK: natural killer
NLRs: nucleotide-binding oligomerization domain-like receptors
Nrf2: nuclear factor E2-related factor 2
NO: nitric oxide
ONOO^-^: peroxynitrite
OXPHOS: oxidative phosphorylation
PLC: phospholipase C
PPAR: peroxisome proliferator-activated receptor
PGC-1: PPARγ coactivator 1
PRR: pattern recognition receptor
qSOFA: quick sequential organ failure assessment
RIPK1: serine/threonine-protein kinase 1
ROS: reactive oxygen species
SFXN1: sideroflexin
SNS: sympathetic nervous system
SOD: superoxide dismutase
STAT1: signal transducer and activator of transcription 1
sIL-6R: soluble IL-6 receptor
SV: stroke volume
TCM: traditional Chinese medicine
TFAM: transcription factor A
TLRs: Toll-like receptors
TNF-α: tumor necrosis factor α
